# Inhibition of Anti-Apoptotic Bcl-2 Proteins in Preclinical and Clinical Studies: Current Overview in Cancer

**DOI:** 10.3390/cells9051287

**Published:** 2020-05-21

**Authors:** Simona D’Aguanno, Donatella Del Bufalo

**Affiliations:** Preclinical Models and New Therapeutic Agents Unit, IRCCS Regina Elena National Cancer Institute, 00144 Rome, Italy; donatella.delbufalo@ifo.gov.it

**Keywords:** cancer, Bcl-2, inhibitors

## Abstract

The dynamic interplay between pro-death and pro-survival Bcl-2 family proteins is responsible for a cell’s fate. Due to the recognized relevance of this family in cancer progression and response to therapy, different efforts have made in recent years in order to develop small molecules able to target anti-apoptotic proteins such as Bcl-2, Bcl-xL and Mcl-1. The limitations of the first Bcl-2 family targeted drugs, regarding on-target and off-target toxicities, have been overcome with the development of venetoclax (ABT-199), the first BH3 mimetic inhibitor approved by the FDA. The purpose of this review is to discuss the state-of-the-art in the development of drugs targeting Bcl-2 anti-apoptotic proteins and to highlight the potential of their application as single agents or in combination for improving anti-cancer therapy, focusing in particular on solid tumors.

## 1. Introduction

Apoptosis is a deeply studied form of programmed cell death that triggers cells to suicide through proteolysis of some key cellular components, which renders cells prone to be recognized by phagocytes [1] The two mechanisms of apoptotic induction are the “death receptor” or “extrinsic” pathway activated by exogenous death-inducing ligands, and the “mitochondrial” or “intrinsic” pathway induced by stress conditions [1]. The intrinsic apoptotic pathway is mediated and regulated by the balance of pro- and anti-apoptotic members of the B cell lymphoma-2 (Bcl-2) family of proteins, and results in alteration of mitochondrial outer membrane permeabilization (MOMP), release of cytochrome c from the mitochondria into cytosol, assembly of the APAF-1 containing apoptosome and activation of the caspase cascade through caspase-9 [2,3]. Bcl-2 family members, characterized by the presence of short conserved sequence regions (Bcl-2 homology [BH] motifs), are classified into three subgroups: the anti-apoptotic/pro-survival proteins, including Bcl-2, Bcl-xL, Mcl-1, Bcl-w, Bcl-B, and A1/Bfl-1; the pro-apoptotic BH3-only proteins, such as Bim, Bid, Bad, Noxa, Puma, and Bmf; and the multidomain pro-apoptotic proteins, such as Bax and Bak [4]. The pro-survival protein subgroup is characterized by the existence of the N-terminal BH4 domain [5]. The BH4 domain of Bcl-2 and Bcl-xL is able to bind other proteins that do not belong to Bcl-2 protein family, allowing them to play a role beyond their classical role in inhibiting apoptosis, in other important cellular functions such as proliferation, autophagy, differentiation, DNA repair, tumor progression, and angiogenesis [6]. The anti-apoptotic Bcl-2 family proteins exert their pro-survival function by binding and inhibiting the pro-apoptotic proteins, the sensors of cellular stress (the BH3-only proteins) and the effectors of apoptosis (Bax and Bak) [7]. From a biochemical point of view the interaction between pro- and anti-apoptotic proteins takes place via the binding of the hydrophobic face of the amphipathic BH3 α-helix from the pro-apoptotic protein into a hydrophobic pocket in the anti-apoptotic protein formed by the BH1, BH2 and BH3 domains [8].

**Figure 1:**
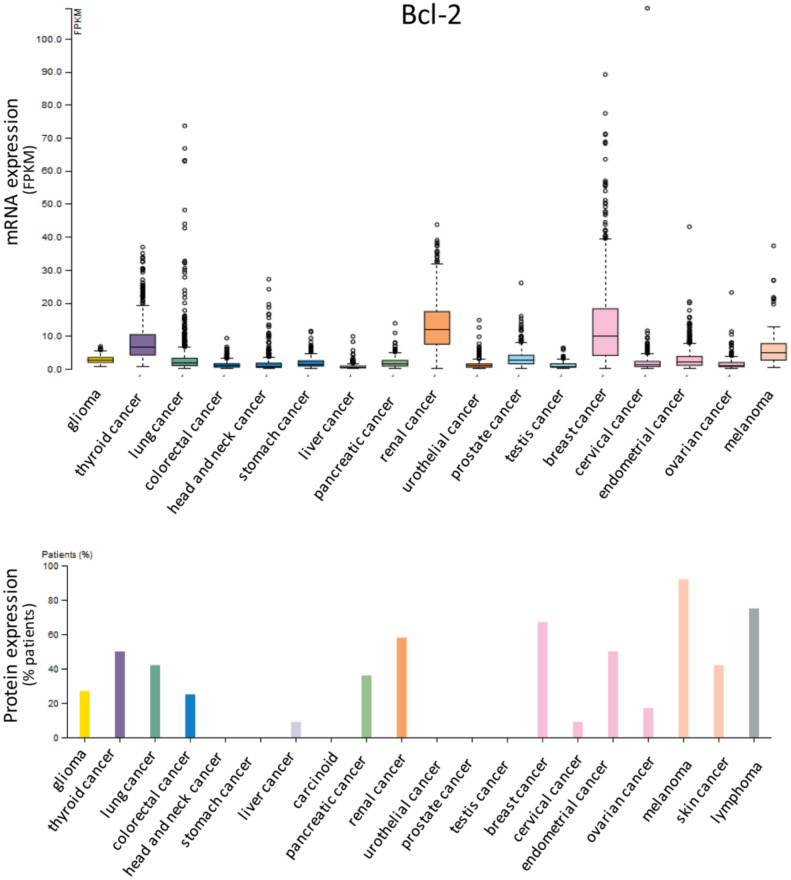
Expression of Bcl-2 in cancer. Bar charts showing the expression in different tumor histotypes of Bcl-2 mRNA, reported as fragments per kilobase of exon model per million reads mapped (FPKM), and protein, detected by immunohistochemistry and reported as percentages of positive patient samples. Data are from The Human Protein Atlas database (https://www.proteinatlas.org/).

**Figure 2:**
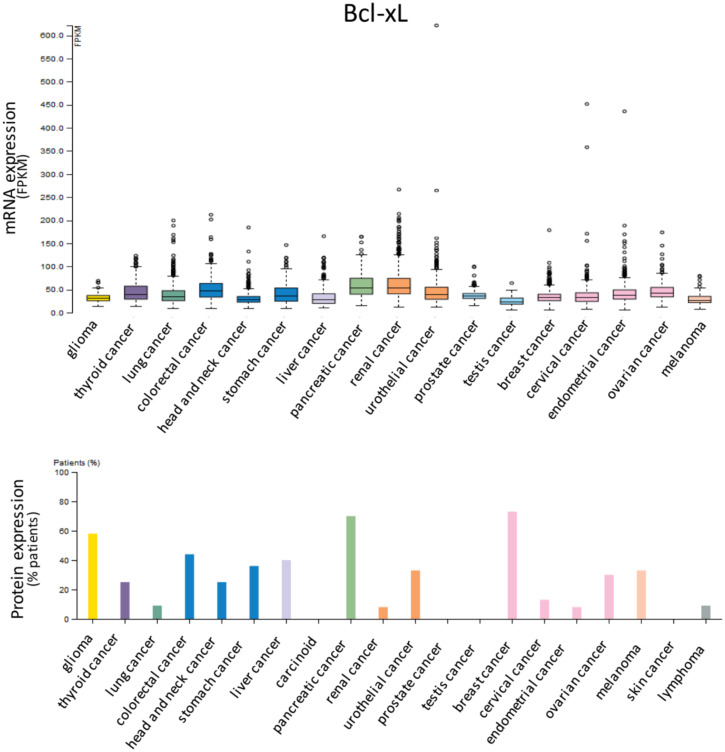
Expression of Bcl-xL in cancer. Bar charts showing the expression in different tumor histotypes of Bcl-xL mRNA, reported as fragments per kilobase of exon model per million reads mapped (FPKM), and protein, detected by immunohistochemistry and reported as percentages of positive patient samples. Data are from The Human Protein Atlas database (https://www.proteinatlas.org/).

**Figure 3:**
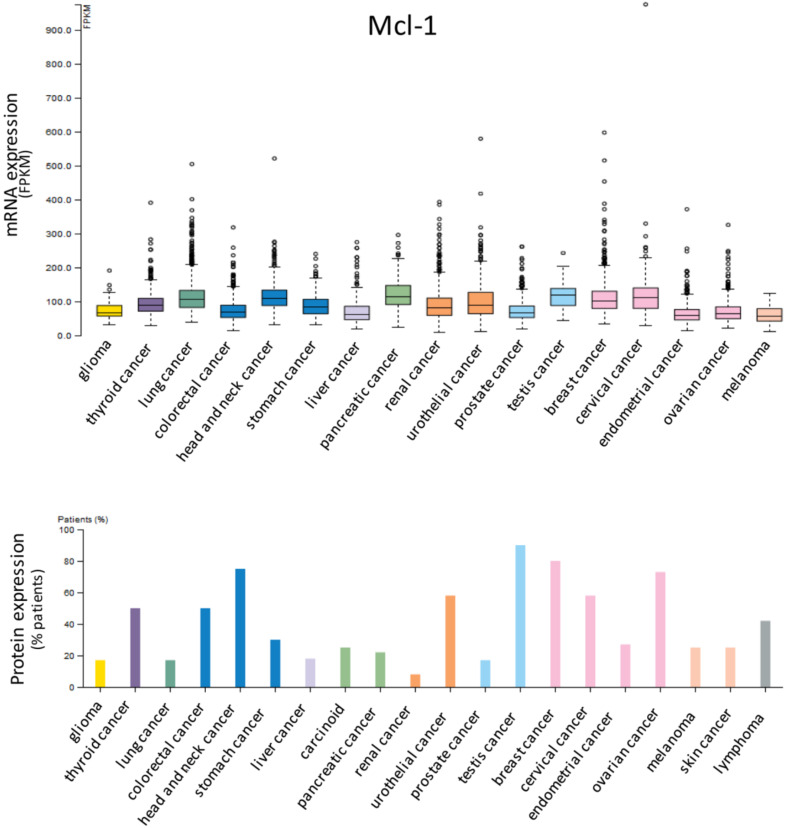
Expression of Mcl-1 in cancer. Bar charts showing the expression in different tumor histotypes of Mcl-1 mRNA, reported as fragments per kilobase of exon model per million reads mapped (FPKM), and protein, detected by immunohistochemistry and reported as percentages of positive patient samples. Data are from The Human Protein Atlas database (https://www.proteinatlas.org/).

Anti-apoptotic proteins Bcl-2 (Figure 1), Bcl-xL (Figure 2) and Mcl-1 (Figure 3) are expressed in a wide range of tumor histotypes, with different extent regarding mRNA and protein levels, by The Human Protein Atlas database (https://www.proteinatlas.org/).

A deregulated expression of several Bcl-2 family members has been observed in cancers from different origin [9]. Moreover, several studies, including ours, have found that high levels of anti-apoptotic proteins contribute not only to modulation of apoptosis and response to chemotherapy, but also to tumor initiation and progression [6,10].

**Figure 4:**
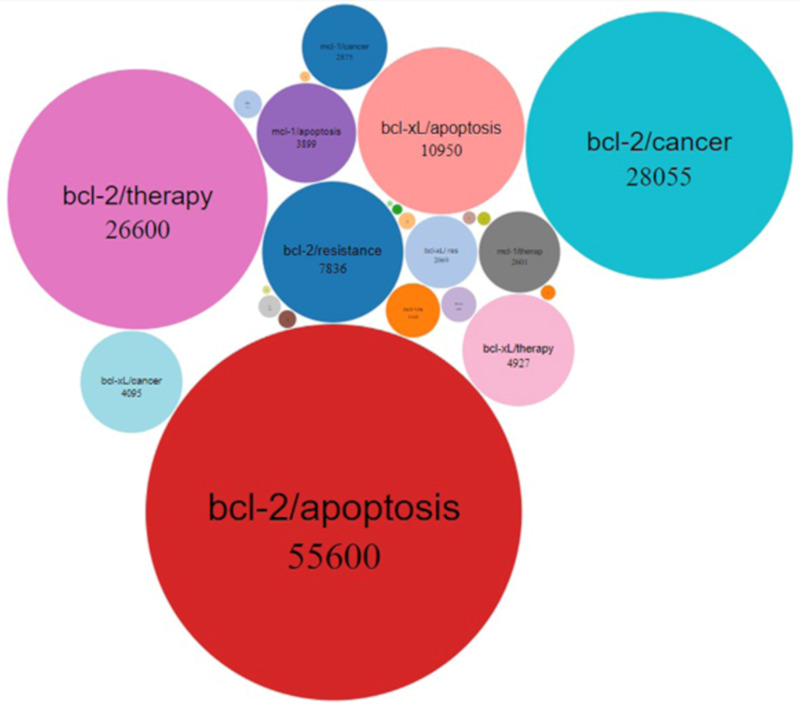
Bubble chart showing the number of published papers in the last 30 years regarding Bcl-2, Bcl-xL, Mcl-1, Bcl-w, Bcl-B and A1/Bfl-1 anti-apoptotic proteins associated with apoptosis, cancer, therapy or resistance.

Since the discovery of Bcl-2, the founding member of the family, many papers have been published regarding the role that Bcl-2 anti-apoptotic members play in cancer and in drug resistance, as well as on their use for cancer therapy. In this regard, a comprehensive representation of the published papers on PubMed (https://pubmed.ncbi.nlm.nih.gov/) since Bcl-2 discovery is reported in Figure 4.

Due to their multiple functions in cancer, Bcl-2 family proteins have become interesting targets for anti-cancer drugs. 

The purpose of this review is to discuss the role of the main Bcl-2 anti-apoptotic proteins in solid cancer, to outline how Bcl-2 family regulation is positioned within the context of cancer treatment and to discuss the potential of Bcl-2 family inhibitors for cancer therapy with the aim of improving patient survival. Given the high volume of information about the relevance of Bcl-2 inhibitors in hematologic malignancies, including acute myeloid leukemia (AML), mature B-Cell malignancies and lymphoid malignancies [11,12,13], this article will focus mainly on Bcl-2 inhibitor application in solid tumors.

## 2. Relevance of Bcl-2 Anti-Apoptotic Family Proteins in Cancer

### 2.1. Bcl-2

The tumorigenic effect of Bcl-2 was first described in subsets of non-Hodgkin’s lymphoma (NHL), such as AML, where it has been found to be associated with chemoresistance and unfavorable outcomes [14,15,16]. Evidence that Bcl-2 may have also oncogenic potential in carcinoma was first provided in prostate cancer, where high expression of the protein was found in androgen-independent tumors [17]. In subsequent studies, increased Bcl-2 expression has been reported in many different solid tumor histotypes, including ovarian [18], breast [19] and lung [20] carcinoma, and melanoma [21,22]. Increased levels of Bcl-2 expression have been also associated with resistance to different drugs including 5-fluorouracil in gastric cancer [23] cisplatin in ovarian cancer [24] and doxorubicin in osteosarcoma and chondrosarcoma [25,26]. In the last years, our group demonstrated that, in addition to its important role in the regulation of apoptosis and chemoresistance [27], Bcl-2 modulates in vitro and in vivo tumor migration, invasion, autophagy and angiogenesis [28,29,30,31,32,33], promotes a cancer stem-like cell phenotype [34], regulates the expression of microRNA and the activity of several transcription factors and their specific target genes [35,36,37], controls an interleukin-1β-driven axis of macrophage diversion that establishes tumor microenvironmental conditions favoring melanoma development [38] and is involved in mitochondrial mRNA homeostasis [39]. We and other authors demonstrated several non-canonical functions of Bcl-2, as well as other anti-apoptotic proteins, in an apoptosis-independent manner [40]. Bcl-2 expression in cancer patient samples is also associated with cancer progression, including liver metastatization in colorectal cancer [41,42], lymphovascular invasion of breast cancer [43,44] and gastric cancer staging [45]. Moreover, Bcl-2 upregulation is particularly evident during the progression from pre-invasive lesions to invasive carcinoma in lung cancer samples [46].

### 2.2. Bcl-xL (BCL2-like 1 Gene, BCL2L1)

Two isoforms of Bcl-X cDNA, Bcl-xL and Bcl-xS, with opposite functions in terms of apoptosis regulation, have been identified. Bcl-xL is an anti-apoptotic protein sharing similar structural domains with Bcl-2, while Bcl-xS, lacking the region with the highest homology to Bcl-2, promotes apoptosis [47]. Different mechanisms are responsible for the alternative splicing, including cellular stress, DNA damage, protein synthesis stalling and protein kinase C inhibition [48]. We and others previously reported the link of Bcl-xL protein expression not only with drug resistance in different tumor histotypes [27]*,* but also with tumor-associated properties, including angiogenesis and cancer cell stemness [34,49,50,51,52]. Recently, it has been demonstrated that Bcl-xL, interacting with Voltage-dependent anion-selective channel 1 through its BH4 domain, favors cell migration by promoting reactive oxygen species in breast cancer models [53]. In tumor patient samples, Bcl-xL upregulation has been reported to correlate with invasion and metastasis in retinoblastoma [54], melanoma [55], breast [56], colon [57], tongue [58] and hepatocellular [59] carcinoma.

### 2.3. Mcl-1 (Myeloid Leukemia Sequence 1)

Mcl-1 was initially discovered in MC-1 hematopoietic cell line were it was found upregulated during differentiation from monocyte to macrophage [60]. High levels of Mcl-1 have been also reported in hematological malignancies and subsequently in a wide range of solid tumors, including breast, ovarian, prostate, pancreatic and non-small cell lung (NSCLC) carcinoma [61,62,63,64,65,66]. Mcl-1 amplification and overexpression are also frequently associated with poor prognosis and resistance to anticancer drugs [67,68,69,70,71,72].

## 3. Anti-Apoptotic Bcl-2 Family Protein Inhibitors

### 3.1. Antisense Oligonucleotides

The first strategy followed in the attempt to inhibit the function of anti-apoptotic Bcl-2 family proteins was to design antisense oligonucleotides directed against the mRNA of the protein of interest. The dual Bcl-2/Bcl-xL and the specific Bcl-xL antisense oligonucleotides were tested by us and other groups in in vitro and in vivo preclinical models [49,73,74,75]. Oblimersen (genasense, G3139), the specific antisense oligonucleotide drug directed against Bcl-2, was the first compound to reach clinical study. After the failure of oblimersen as a single agent, its efficacy in combination with other drugs was evaluated in several Phase I–III clinical trials in patients with advanced solid malignancies, but they were discontinued [76,77,78,79]. A list of completed clinical trials with oblimersen is reported in Supplementary Table S1.

### 3.2. BH3 Mimetics

**Figure 5:**
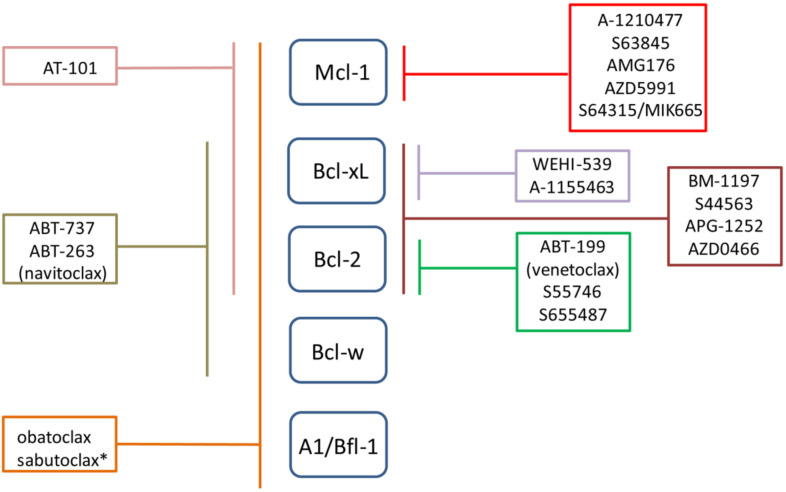
Schematic representation of BH3 mimetics. For each BH3 mimetic the corresponding Bcl-2 anti-apoptotic protein targets are indicated by lines categorizing BH3 mimetics according to their specificity (multitargets, dual or specific inhibitors). * Sabutoclax is not reported to inhibit Bcl-w.

In the past decades, different efforts have been made in order to understand the network of protein-protein interactions involved in the regulation of apoptosis mediated by Bcl-2 family members. The understanding of the interaction among Bcl-2 family members has been the foundation of drug discovery approaches, based on innovative medicinal chemistry and structure-based drug design, with the aim of generating small-molecule inhibitors of anti-apoptotic Bcl-2 family proteins, which mimic the function of the BH3-only proteins to kill cancer cells [80]. The BH3 mimetics class of inhibitors is mainly represented by molecules with low level of specificity and high affinity for different anti-apoptotic Bcl-2 proteins, although in recent years specific Bcl-2 protein inhibitors have been developed. A schematic list of BH3 mimetics is reported in Figure 5.

Despite significant efforts, ten BH3-mimetic drugs (obatoclax, AT-101, ABT-263 (navitoclax), APG-1252, AZD0466, venetoclax, S55746, AMG-176, AZD5991 and S64315/MIK665) have reached clinic with only the Bcl-2 inhibitor venetoclax currently approved by FDA [81,82].

#### 3.2.1. Rationale for the Use of BH3 Mimetics (Priming and Protein Addiction)

Cancer cell dependency on specific anti-apoptotic Bcl-2 proteins could be explained by multiple factors, including tissue of origin, impact of the oncogenic lesions that drove tumorigenesis, and/or factors produced by the tumor stroma [82]. Anti-apoptotic proteins are often expressed at high levels in cancer cells, forming high numbers of complexes with their pro-apoptotic counterparts, a condition described by the concept of “priming” [8]. Primed cancer cells are more sensitive to BH3 mimetics (and other anti-cancer agents) compared with their “normal” counterparts [8]. The relative expression levels between anti-apoptotic Bcl-2 family members and pro-apoptotic BH3 only proteins were found to correlate with sensitivity to BH3-mimetic drugs [83].

The “protein addiction” phenomenon, the dependence of response to drugs in tumor cells on the expression level of members of an anti-apoptotic family, is mostly linked to a single pro-survival protein in leukemia and lymphoma, while in solid tumors it is often associated with multiple anti-apoptotic protein levels [82,84]. Dependencies of tumor cells on anti-apoptotic Bcl-2 family members can be experimentally determined by the so-called “dynamic BH3 profiling”, where BH3 peptides specific for individual BH3-only proteins are applied to permeabilized cells and allowed to interact with other BH3-containing proteins on the surface of the mitochondria, generating MOMP, after allowing Bax or Bak oligomerization [8]. Other experimental approaches could be the use of inducible CRISPR/Cas9 platform [85] or culturing malignant cells from the patient with different BH3-mimetic drugs [86].

#### 3.2.2. Multitarget BH3 Mimetics

The first generation of BH3 mimetics had limited selectivity for a specific anti-apoptotic Bcl-2 protein and most of them were also found to promote cell death independently of Bax/Bak proteins. The pan BH3 inhibitor obatoclax (GX15-070), showing affinity for Bcl-2, Bcl-xL, Mcl-1, Bcl-w, A1/Bfl-1 [87] and the R-(-) enantiomer of gossypol acetic acid, AT-101, able to bind to Bcl-2, Bcl-xL and Mcl-1 [88], was were evaluated in Phase I/II clinical trials in hematological malignancies and in solid tumors, including small-cell lung cancer (SCLC) and metastatic melanoma in the case of obatoclax (listed in Supplementary Table S2) and lung cancer, prostate cancer, squamous cell carcinoma of the head and neck and brain and central nervous system tumors in the case of AT-101 (listed in Supplementary Table S3). Due to significant toxicities associated with off-target effects, further development of both obatoclax and AT-101 was halted [89,90].

Preclinical studies demonstrated that sabutoclax (BI-97C1), an apogossypol derivative and pan-active Bcl-2 protein family antagonist (inhibiting Bcl-2, Bcl-xL, Mcl-1 and A1/Bfl-1), overcame drug resistance, eliminated cancer stem cells in breast cancer [91] and synergized with minocycline, a synthetic tetracycline, in a pancreatic cancer model [92] and with docetaxel in a model of prostate cancer [93].

ABT-737 was one of the pioneer BH3 mimetics [94]. It is a small-molecule inhibitor of Bcl-2, Bcl-xL and Bcl-w resulting from the combination of nuclear magnetic resonance-based screening, parallel synthesis and structure-based design. The mechanism of action was demonstrated in chronic lymphocytic leukemia (CLL) cells [95], but its efficacy and synergistic cytotoxicity with chemotherapeutics and radiation was also reported for solid tumors including SCLC [94]. In preclinical models of lung cancer, ABT-737 in combination with the inhibition of Notch by the use of GSI, a γ-secretase inhibitor, showed a synergistic antitumor effect in vitro and significantly suppressed tumor proliferation compared to the single drug treatment in vivo [96]. Studies conducted in melanoma models demonstrated the ability of ABT-737 to empower the efficacy of several therapeutic strategies including immunotoxins [97] and BRAF or MEK inhibitor in BRAF-mutated cells [98].

Being not orally bioavailable, ABT-737 development has been limited, and navitoclax, its orally bioavailable analog, has been developed. Navitoclax showed efficacy in vivo in xenograft models of leukemia and lymphoma [99] and in vitro in the treatment of SCLC cells [100]. Combination of navitoclax with PI3K inhibition suppressed tumor growth in both an established SCLC xenograft model and in an established circulating tumor cell-derived explant model generated from a blood sample obtained at presentation from a chemorefractory SCLC patient [101]. The efficacy of navitoclax has been reported for treatment of BRAFV600E positive in vivo melanoma models in combination with copper chelators, able to sequester copper required for MEK1 and MEK2 activity through a direct copper-MEK1/2 interaction [102].

Navitoclax effectiveness was limited by dose-dependent Bcl-xL-mediated thrombocytopenia [103]. In order to reduce the toxicity of navitoclax, new technology has been employed, converting navitoclax into DT2216, a Bcl-xL proteolysis-targeting chimera (PROTAC) that targets Bcl-xL to the Von Hippel-Lindau (VHL) E3 ligase for degradation. Since VHL is little expressed in platelets, the toxicity in these cells is reduced, while the therapeutic potential of DT2216 remains similar to those of the original molecule when evaluated in several xenograft tumors as a single agent or in combination with other chemotherapeutic agents [104].

Given the relevant role of proteins of the Bcl-2 family in regulating clonal selection and survival of lymphocytes, and their frequent overexpression in lymphomas, navitoclax progressed to clinical evaluation, firstly as single agent Phase I/II trial in patients with this malignancy [105]. Regarding its efficacy in solid tumors, navitoclax was shown to synergize with several chemotherapeutics such as doxorubicin [106] and paclitaxel [107] in triple-negative breast cancer models, with PARP inhibitor in high-grade serous ovarian cancer [108] and with TORC1/2 inhibitor in colorectal cancer [109].

At present, navitoclax is under clinical evaluation in combination with ruxolitinib, a JAK inhibitor, in myeloproliferative neoplasm (NCT04041050), myelofibrosis (NCT03222609) and lymphoid cancers (NCT00788684). Clinical trials are also ongoing using navitoclax in combination with osimertinib, an epidermal growth factor receptor tyrosine kinase inhibitor, in NSCLC (NCT02520778); sorafenib, a kinase inhibitor, in relapsed or refractory (R/R) solid tumors (NCT02143401); trametinib, a MEK inhibitor, in advanced or metastatic solid tumors (NCT02079740); and dabrafenib (a BRAF inhibitor)/trametinib in BRAF mutant melanoma (NCT01989585). Active clinical trials with BH3 mimetics are reported in Table 1.

#### 3.2.3. Dual BH3 Inhibitors: Bcl-2/Bcl-xL, Bcl-2/Mcl-1, Bcl-xL/Mcl-1

Starting from the arylsulfonamide scaffold of ABT-737/ABT-263, several dual Bcl-2/Bcl-xL inhibitors have been generated. BM-1197, in SCLC models, has been demonstrated to have potent in vitro proliferation inhibitory effect and to achieve in vivo complete and long-term tumor regression in xenograft models, associated with reversible platelet reduction at highly efficacious doses [110]. BM-1197 efficacy was also tested in human colorectal cancer, where it increased the fraction of cells in the sub-G1 phase of the cell cycle, induced apoptotic death and increased the cellular inter nucleosomal DNA fragmentation [111]. Furthermore, in malignant lymphoma cells BM-1197 induced cell death through the intrinsic apoptotic pathway [112].

S44563 was found to enhance in vitro and in vivo radiosensitivity of SCLC cells [113] and to increase the efficacy of fotemustine, a nitrosourea alkylating agent, in uveal melanoma patient-derived xenografts [114].

APG-1252 (BM-1252), a recently developed drug with high binding affinity to Bcl-2 and Bcl-xL, has been reported to induce mitochondria-dependent apoptosis in leukemia cells in vitro, to achieve complete and persistent tumor regression in multiple tumor xenograft models including ALL, SCLC and colon and breast carcinoma, and shows strong synergy with some chemotherapeutic agents [115,116]. Phase I trials with APG-1252 alone or in combinatorial therapy are currently ongoing for the treatment of patients with lung carcinoma or other solid tumors (NCT03080311, NCT04210037). Moreover, AZD0466, a dual Bcl-2/Bcl-xL inhibitor is under clinical evaluation in hematologic malignancies and advanced solid tumors (NCT04214093). Clinical trials with BH3 mimetics are reported in Table 1.

Single agent antitumor activity of S1, the BH3-mimetic dual inhibitor of Bcl-2 and Mcl-1, and its derivative B4, has been also reported in cancer models with different origin [117].

Starting from the natural compound meiogynin A, molecules specifically targeting Bcl-2/Mcl-1 [118] or Bcl-xL/Mcl-1 [119] have been synthesized and in vitro tested, nevertheless with limited application until now. Moreover, two PROTACs compounds have been recently developed for the selective degradation of Mcl-1/Bcl-2 [120].

The combination of BH3 mimetics targeting different anti-apoptotic proteins such as Bcl-2 and Mcl-1 or Bcl-xL and Mcl-1, has been reported to shown significant benefit for the treatment of melanoma [121].

#### 3.2.4. Bcl-2 Specific Inhibition

The turning point in the research for Bcl-2 inhibitors was reached with the development of venetoclax, a potent and selective BH3 mimetic for Bcl-2 protein, which was able to circumvent the thrombocytopenia observed with navitoclax [122]. The first FDA approval for venetoclax in first-line treatment of patients with the R/R CLL and carrying the 17p deletion came in 2016 [123]. Subsequently, in June 2018, the clinical practice of venetoclax was introduced by the FDA for patient with CLL or small lymphocytic lymphoma, regardless of 17p deletion. After the first excellent findings, venetoclax was tested in combination, with satisfactory results, in different hematological malignancies [11,12,13,124]. In November 2018, the FDA approved the use of venetoclax in combination with hypomethylating drugs azacitidine, decitabine or low-dose cytarabine for the treatment of newly-diagnosed AML in adults who are aged 75 years or older, or who have comorbidities that preclude use of intensive chemotherapy. In January 2019, the impressive results of the Murano Phase III trial [125] prompted the European Commission to approve the combination of venetoclax/rituximab for patients with R/R CLL, previously treated without success. Other combination therapies have been evaluated in clinical trials in hematological tumors. In CLL, venetoclax was evaluated in combination with obinutuzumab, an anti-CD20 monoclonal antibody, in Phase Ib [126], or with ibrutinib, an inhibitor of Bruton’s tyrosine kinase, in Phase II [127,128]. Venetoclax showed activity in a Phase I trial in multiple myeloma (MM) patients carrying t(11;14) with multiple prior lines of therapy as single agent [128] and in combination with bortezomib, a proteasome inhibitor, and dexamethasone in a Phase Ib study [129]. In a small cohort of patients affected by R/R AML and treated with venetoclax as single agents, subjects with isocitrate dehydrogenase (IDH) 1/2 mutations were found to have better response in respect to patients carrying the wild type gene [130,131]. Venetoclax in combination with navitoclax and chemotherapy is under evaluation in subjects with R/R ALL or R/R LL (NCT03181126). Mechanisms of resistance to venetoclax have been identified in G101V-mutated Bcl-2 proteins in CLL patients [132], thus emphasizing the need to persevere in research of Bcl-2 inhibitors.

In 2019, the first clinical study evaluating the efficacy of venetoclax in solid tumors demonstrated that the combination of venetoclax with tamoxifen showed a tolerable safety profile and activity in estrogen-receptor and Bcl-2-positive metastatic breast cancer [133].

Several trials are ongoing to evaluate the potential of the combination therapy of venetoclax in advanced solid tumors, in particular in combination with ABBV-181, an anti-PD1 monoclonal antibody (NCT03000257), with ABBV-621, a second-generation TRAIL-receptor agonist (NCT03082209), and with idasanutlin, a small molecule designed to bind to murine double minute 2 (MDM2), for pediatric and young adult patients with neuroblastoma or other malignancies (NCT04029688).

Other BH3 mimetics targeting Bcl-2 such as S55746 [134] and its prodrug S65487 are under evaluation in clinical trials for the treatment of CLL, NHL and MM patients (NCT03755154). Active clinical trials with specific BH3 mimetics are reported in Table 1.

Several preclinical studies also investigated the efficacy of venetoclax in combination therapy for solid tumors. Studies conducted in melanoma models demonstrated the ability of venetoclax to empower the efficacy of mitochondrial matrix chaperone inhibitor [135]. Studies performed in estrogen-positive breast cancer cell lines, patient-derived organoid and patient-derived xenograft models evidenced the efficacy of venetoclx in combination with both fulvestrant/palbociclib and anti-PD1 therapy [136], while results on merkel cell carcinoma indicated the combination of venetoclax with DNA damage induction as a possible novel therapeutic strategy for this skin cancer [137].

Resistance to venetoclax in NHL cell lines has been reported to be overcome by Mcl-1 selective inhibitor A-1208746 or Bcl-xL selective inhibitor A-1155463 in combination with venetoclax [138].

Venetoclax and navitoclax synergized with doxorubicin or dinaciclib, an inhibitor of cyclin-dependent kinases, providing effective therapeutic strategies in SCLC [84].

#### 3.2.5. Bcl-xL Specific Inhibition

Experimental evidence indicates that overexpression of Bcl-xL could be associated with resistance to venetoclax and chemotherapic agents [138,139,140]. This consideration prompted the development of specific Bcl-xL inhibitors. WEHI-539 was the first molecule to specifically target Bcl-xL [141]. It was found to enhance apoptosis in combination with doxorubicin in osteosarcoma cells expressing high level of Bcl-xL protein [142]. However, the observed in vivo toxicity limited its applicability in clinical trials [141]. Structure-based design optimization of WEHI-539 led to the development and characterization at preclinical level of other selective inhibitors of Bcl-xL, such as A-1155463, showing in vivo antitumor activity in a xenograft model of SCLC, and A-1331852 increasing the sensitivity of rhabdomyosarcoma cells to several conventional chemotherapeutic agents without apparent toxicity [139,143,144]. To date, no specific Bcl-xL inhibitor has passed to clinical evaluation.

#### 3.2.6. Mcl-1 Specific Inhibition

In recent years an increasing interest in developing a Mcl-1-specific inhibitor is arising, especially for hematological tumors. Rationale and progress in targeting Mcl-1 in hematologic malignancies have been recently reviewed [145].

UMI-77 was the first designed molecule with selected affinity for Mcl-1 [146], showing in vivo tumor growth inhibition in models of pancreatic cancer. Subsequent developed molecules were A-1210477, showing cell killing activity as single agents and in combination with navitoclax in leukemia cells [147], and inducing apoptosis in breast cancer [148], as well as compounds 4 and 5, showing apoptosis activity in primary MM and AML patient-derived cells [149].

S63845 showed high level of apoptotic induction in a wide range of hematopoietic malignancies, breast and NSCLC cell lines and with an acceptable safety margin as a single agent in in vivo experiments in several cancer models [86]. Several preclinical studies suggested the promising application of Mcl-1 inhibitors in combinatorial therapy: combinations of S63845 and AMG-176 (and the related compound AM8621) with inhibitors of fibroblast growth factor receptor (FGFR), MEK or BRAF showed to efficiently reduce in vitro cell proliferation and in vivo tumor growth of NSCLC [86,150], lung squamous cell carcinoma [151] and glioblastoma [152] models, while combinations of S63845 with chemotherapy or HER2-targeted therapies demonstrated to efficiently inhibit the growth of triple-, as well as HER-2-positive breast cancer [86,153]. The efficacy of combination of S63845 with selective inhibitors, such as ABT-199 or A-1331852, has been also reported in preclinical cervical cancer models [154] and pediatric solid tumors [155], while S63845 in combination with inhibitors of the bromodomain and extra-terminal proteins showed efficacy in metastatic melanoma [156].

The first Mcl-1 inhibitor to progress in Phase I trial was AMG-176, evaluated in R/R MM and R/R AML (NCT02675452). Unfortunately, some clinical trials using Mcl-1 inhibitors, such as AMG-397, have been suspended for cardiac toxicity (NCT03465540). This toxicity can be due to the relevant role of Mcl-1 in cardiac homeostasis in adult murine models, loss of Mcl-1 causing alterations in cardiomyocytes and lethal cardiomyopathy [157,158].

AZD5991 and S64315/MIK665 at present are under evaluation in clinical trials as a single agent, Phase I, or in combination with venetoclax, Phase II, in R/R hematologic malignancies (NCT03218683, NCT02979366, NCT02992483, NCT03672695). Clinical trials for evaluation of Mcl-1 BH3-specific inhibitors are reported in Table 1.

Recently, VU661013, a new Mcl-1-specific inhibitor, has been reported to rescue venetoclax resistance in AML [159] and inhibit cell survival in estrogen receptor-positive breast cancer when used in combination with navitoclax [160].

An alternative strategy to the use of specific Mcl-1 inhibitors is to target Mcl-1 indirectly. Since Mcl-1 expression is regulated by the transcriptional activator Cyclin-dependent kinase 9 (CDK9), the use of specific CDK9 inhibitors is a promising approach for the treatment of tumors expressing high levels of Mcl-1 protein [161]. The highly selective CDK9 inhibitor, AZD4573, induced apoptosis and subsequent cell death in hematologic cancer models in vitro and favored tumor regression in tumor xenografts in vivo, through the indirect inhibition of Mcl-1 [161]. AZD4573 is currently under evaluation in a Phase I clinical trial for patients with hematologic malignancies (NCT03263637).

### 3.3. BH3 Peptides

In addition to molecules resembling the BH3 domain, the use of BH3 peptides has also been evaluated as a possible strategy to inhibit Bcl-2 anti-apoptotic members. Biochemical studies based on computational mutagenesis and docking approaches led to the development of BINDI proteins, formed by a BH3-like central helix with two flanking regions which form additional interactions that are specific for the different anti-apoptotic Bcl-2 proteins. This strategy resulted in proteins able to bind anti-apoptotic Bcl-2 proteins with increased affinity and specificity, and demonstrated that the designed inhibitors were able to induce apoptosis in cancer cells in vitro by engaging the BH3-binding grooves of specific pro-survival proteins [162].

A very recent and elegant paper explored the possibility of de novo designing switchable protein systems to modulate binding among proteins, and demonstrated the possibility to modulate the binding of Bcl-2 to Bim, thus opening the prospective of using this system to regulate the function of Bcl-2 family members [163].

A series of BH3 sensitizer peptides that bind Bcl-xL with sub-nanomolar affinity and selectivity up to 1000-fold over each of the competing pro-survival proteins, have been tested in vitro in a panel of cancer cell lines, showing reduced proliferation in cells expressing high level of Bcl-xL protein [164].

### 3.4. Molecules Promoting Protein Conformational Change

Another interesting approach to inhibit Bcl-2 functions is to target the BH4 domain. The BH4 domain of Bcl-2 binds to the inositol 1,4,5-trisphosphate receptor (IP3R), preventing Ca2+ signals that mediate cell death. In many cancers the high level of Bcl-2 expression inhibits IP3R-mediated Ca2+ elevation, thus preventing apoptosis [165]. Moreover, the BH4 domain has been also reported to be involved in the so-called “non-canonical” Bcl-2-mediated functions, not including anti-apoptotic and pro-survival functions [6]. Thus, BH4 has become an interesting target for drug development [166]. Structural studies were employed to identify BDA-366, an allosteric Bcl-2 inhibitor able to bind the BH4 domain, inducing a conformational change in the protein responsible for the exposure of the BH3 domain, which converts Bcl-2 in a pro-apoptotic protein [167]. BDA-366 was found to inhibit both lung cancer [167] and myeloma growth in vitro and in vivo [168].

The Zhang group identified the Q221R222N223 motif, QRN, as a hidden conformational switch controlling ubiquitination of Mcl-1, and demonstrated the ability of compound 5, a dual-function Mcl-1 inhibitor, to favor Mcl-1 ubiquitination by promoting helical conformation of QRN, thus inducing both apoptosis and Mcl-1 degradation [169].

A new approach for disarming Mcl-1 in cancer identified an allosteric mechanism able to disrupt the binding activity of Mcl-1 to BH3 domain of pro-apoptotic proteins: allosteric Mcl-1 inhibitors specifically target Cysteine 286, inducing conformational changes and allosteric inhibition of BH3 domain interaction with Mcl-1 [170], or bind to Lysine 234 allowing specific increase in binding to Mcl-1 over other Bcl-2 family members [171].

### 3.5. Bcl-2 Quadruplex Selective Approach

Preclinical evidence also supports the Bcl-2 G-quadruplex (G4)-selective approach to treat cancer and to circumvent the limitations of Bcl-2 protein-based therapeutics [172]. In the proximity of the P1 promoter (5′), three G4 and one i-motif have been reported to regulate Bcl-2 transcription [173]. Furthermore, the Bcl-2 5′ untranslated region containing an RNA G4-forming motif has been found to modulate Bcl-2 protein expression [174]. The in-depth characterization of these structures provided information for designing small molecules targeting G4 and regulating the expression of Bcl-2. The desirable molecules should show high affinity to Bcl-2 G4 and low affinity to duplex or other G4. Although a high number of G4, mainly identified by computational methods or structure-based drug design, show good binding affinity to the Bcl-2 quadruplex, they could not achieve good drug candidature for their failure to discriminate different G4, such as telomeric and other oncogenic G4. At present only a few compounds targeting G4 structures have been successfully evaluated in cellular and in vivo models. Quindoline, perylene and coronenen derivatives have been reported to downregulate Bcl-2 transcription and promoter activity and to induce apoptosis in cancer models but with a promiscuous mechanism of action affecting also other biological targets, such as telomeric and other oncogenic G4 [175,176]. Some organometallic complexes have been found to be more selective to Bcl-2 than to telomeric quadruplex, to have poor affinity for duplex DNA and to exhibit in vitro and in vivo antitumoral activity against cancer models from different histotypes [177]. Very recently, drug-like imidazo [2,1-i] purine derivatives have been identified by a bioinspired design and have been reported to show antitumoral activity through their effect on Bcl-2/c-myc gene promoter G4 [178]. Furthermore, furopyridazinone-based molecules have been found to target the Bcl-2 gene promoter G4 with good selectivity and induce cytotoxic effect in T-lymphoblastoid cells [179]. Ligands able to target and stabilize G4 structures both in the Bcl-2 gene and in its RNA transcript have been also reported [180]. Due to the promiscuity to other molecular targets, to the poor bioavailability and to the conformational rigidity, the clinical pharmacology of G4-stabilizing molecules is still at the beginning.

## 4. Vaccination Using Anti-Apoptotic Protein-Derived Peptides

The induction of active immunity against tumor-associated antigens may be a promising approach to prevent cancer relapse, and thus there is a need to identify tumor-associated antigens for the development of cancer vaccination. In this context, spontaneous T cell responses against anti-apoptotic protein-derived peptides in patients suffering from cancers of different origin have been reported [181]. In particular, in vitro T cell responses against a peptide derived from Bcl-xL was observed in cancer patients but not in healthy controls, and the subpopulation of T cells specific for the Bcl-xL peptide was cytotoxic against HLA-matched cancer cells of different histotypes [181]. Two murine tumor-associated epitopes derived from mouse Bcl-xL have been reported to induce CD8+ T cell production of interferon-γ in mice, providing a preclinical model for cancer vaccination [182]. These preclinical studies supported the passage to Phase I trial evaluation of therapeutic vaccination with peptides from Bcl-2, Bcl-xL and Mcl-1 in patients with relapse MM (NCT01272466). In this study, vaccines were given in combination to treatment with bortezomib [183]. This vaccination was well tolerated and the signs of toxicity were all attributed to bortezomib. Moreover, the safety, toxicity and immunological effect of vaccine Bcl-xl_42-CAF09b, composed of Bcl-xl_42, a peptide fragment of the full protein and the adjuvant CAF09b, able to improve the activation of the immune system, are under evaluation in patients with hormone-sensitive prostate cancer and lymph node metastases in a Phase I trial (NCT03412786).

## 5. Pro-Apoptotic Bcl-2 Family Protein Activations

Several structural studies have helped the understanding of determinant for regulation and activation of pro-apoptotic proteins [184,185]. These findings have been used to design peptides specifically targeting and activating the pro-apoptotic function of Bax, Bak and Bim, in order to promote cell death [186].

The use of a hydrocarbon-stapled Bim BH3 peptide (Bim SAHBA) was shown to overcome both Bcl-2 and Mcl-1 apoptotic resistance in B-cell lymphoma cell lines [187].

In another study, the potent BH3 α-helical domain of Bim has been incorporated into peptide amphiphile nanostructures to facilitate cellular uptake, showing specificity of binding to Bcl-2 anti-apoptotic proteins and inducing cell death in mouse embryonic fibroblasts [188].

Computational methods have been applied in order to design BH3 peptides derived from Puma and Bmf showing high binding affinity for A1/Bfl-1 [189].

Free energy binding studies of complexes formed by Bak and BH3 peptides have been employed to find the main residues responsible for inhibition of activation of Bak, which are useful in designing novel small molecule mimics of BH3 able to promote the mitochondrial pore formation mediated by Bak [190].

Although Bcl-2 is a recognized anti-apoptotic protein, in some conditions Bcl-2 associates with the orphan nuclear hormone receptors Nur77 and Nor-1, converting Bcl-2 into a pro-apoptotic molecule [191]. NuBCP-9, a Nur77-derived peptide, induces a conformational change, exposing the Bcl-2 BH3 domain, finally inhibiting tumor growth in vitro and in vivo [192].

## 6. Conclusions

The involvement of apoptosis has been long studied for its response to conventional chemotherapy, but its relevance is also clear in response to more innovative treatment strategies. The family of Bcl-2 proteins has long been known to play a pivotal role in the regulation of apoptosis. In the last decades, a huge amount of evidence has demonstrated that cancers from different origin, especially hematological malignancies, strictly depend on anti-apoptotic members for proliferation, survival and response to therapy. In this view, intense studies have been performed in order to identify Bcl-2 inhibitors to be used for cancer therapy, and cell death discoveries have been translated into the identification of novel therapies using Bcl-2 family inhibitors. Starting from the clinical use of antisense oligonucleotides directed against Bcl-2, and passing through BH3 mimetics that showed severe on-target toxicity, recent FDA approval of the BH3 mimetic venetoclax corroborated the clinical relevance of using Bcl-2 anti-apoptotic members as therapeutic targets, not only for hematologic malignancies but also for breast carcinoma. Numerous clinical trials are ongoing to evaluate the activity on solid tumors of specific or dual inhibitors as single agents or in combination therapy. We hope positive results can offer a way for these therapeutic strategies to be used for treatment of a large amount of solid malignancies.

A huge volume of preclinical studies have accumulated evidence in support of the role played by anti-apoptotic proteins in the progression of solid tumors. Thus, the use of Bcl-2 protein-targeting drugs as single agents or in combination with current standard-of-care therapies could represent a concrete opportunity to overcome therapy-resistant/recurrent solid tumors and to increase the disease-free survival of cancer patients. Further studies are required to confirm the clinical potential of Bcl-2 inhibitors as single and combinatorial agents for the therapy of chemotherapy-sensitive and resistant cancer. It is likely that a number of malignant diseases other than hematologic ones in the near future will be successfully targeted with anti-apoptotic Bcl-2 family members after careful patient selection, to improve treatment responses and patient survival.

## Figures and Tables

**Table 1:** Active clinical trials with BH3 mimetics (update 7 April 2020).

Inhibitor	Specificity	Clinical Trial Identifier	Tumor Histotype	Phase	Combination with
Navitoclax	Bcl-2/Bcl-xL/ Bcl-w	NCT04041050	myelo- proliferative neoplasm	I	Ruxolitinib or single agent
		NCT03222609	myelofibrosis	II	Ruxolitinib or single agent
		NCT00788684	lymphoid cancers	I	Ruxolitinib
		NCT02520778	advanced or metastatic non-small lung cancer	I	Osimertinib
		NCT02143401	relapsed or refractory solid tumors	I	Sorafenib
		NCT02079740	advanced or metastatic solid tumors	I/II	Trametinib
		NCT01989585	BRAF mutant melanoma	I/II	Dabrafenib/ Trametinib
APG-1252	Bcl-2/Bcl-xL	NCT03080311	small cell lung cancer or other solid tumors	I	single agent
		NCT04210037	relapsed/ refractory small cell lung cancer	I/II	Paclitaxel
AZD0466	Bcl-2/Bcl-xL	NCT04214093	hematologic or solid tumors	I	single agent
Venetoclax	Bcl-2	NCT03000257	advanced solid tumors	I	ABBV-181
		NCT03082209	previously treated solid tumors and hematologic malignancies	I	ABBV-621
		NCT03181126	relapsed/ refractory acute lymphoblastic leukemia and relapsed/ refractory lymphoblastic lymphoma	I	Navitoclax
		NCT04029688	relapsed/refractory acute leukemias or solid tumors	I/II	Idasanutlin
S65487	Bcl-2	NCT03755154	acute myeloid leukemia, non-Hodgkin lymphoma or multiple myeloma	I	single agent
AMG-176	Mcl-1	NCT02675452	relapsed or refractory multiple myeloma and subjects with relapsed or refractory acute myeloid leukemia	I	single agent
AZD5991	Mcl-1	NCT03218683	relapsed or refractory hematologic malignancies	II	Venetoclax
S64315/ MIK665	Mcl-1	NCT02979366	acute myeloid leukemia or myelodysplastic syndrome	I	single agent
		NCT02992483	refractory or relapsed lymphoma or multiple myeloma	I	single agent
		NCT03672695	acute myeloid leukemia	I	Venetoclax

## Supplementary Materials

The following are available online at https://www.mdpi.com/2073-4409/9/5/1287/s1, Table S1: List of completed clinical trials for the evaluation of oblimersen; Table S2: List of completed clinical trials for the evaluation of obatoclax; Table S3: List of completed clinical trials for the evaluation of AT-101.

## References

[1] Hotchkiss R.S., Strasser A., McDunn J.E., Swanson P.E. (2009). Cell death. N. Engl. J. Med..

[2] Tait S.W., Green D.R. (2010). Mitochondria and cell death: Outer membrane permeabilization and beyond. Nat. Rev. Mol. Cell Biol..

[3] Yuan S., Akey C.W. (2013). Apoptosome structure, assembly, and procaspase activation. Structure.

[4] Luna-Vargas M.P., Chipuk J.E. (2016). The deadly landscape of pro-apoptotic BCL-2 proteins in the outer mitochondrial membrane. FEBS J..

[5] Garcia-Saez A.J. (2012). The secrets of the Bcl-2 family. Cell Death Differ..

[6] Gabellini C., Trisciuoglio D., Del Bufalo D. (2017). Non-canonical roles of Bcl-2 and Bcl-xL proteins: Relevance of BH4 domain. Carcinogenesis.

[7] Kale J., Osterlund E.J., Andrews D.W. (2018). BCL-2 family proteins: Changing partners in the dance towards death. Cell Death Differ..

[8] Montero J., Letai A. (2018). Why do BCL-2 inhibitors work and where should we use them in the clinic?. Cell Death Differ..

[9] Hata A.N., Engelman J.A., Faber A.C. (2015). The BCL2 Family: Key Mediators of the Apoptotic Response to Targeted Anticancer Therapeutics. Cancer Discov..

[10] Adams J.M., Cory S. (2018). The BCL-2 arbiters of apoptosis and their growing role as cancer targets. Cell Death Differ..

[11] Yogarajah M., Stone R.M. (2018). A concise review of BCL-2 inhibition in acute myeloid leukemia. Expert Rev. Hematol..

[12] Jullien M., Gomez-Bougie P., Chiron D., Touzeau C. (2020). Restoring Apoptosis with BH3 Mimetics in Mature B-Cell Malignancies. Cells.

[13] Fletcher L., Nabrinsky E., Liu T., Danilov A. (2020). Cell Death Pathways in Lymphoid Malignancies. Curr. Oncol. Rep..

[14] Cassier P.A., Castets M., Belhabri A., Vey N. (2017). Targeting apoptosis in acute myeloid leukaemia. Br. J. Cancer.

[15] Vitagliano O., Addeo R., D’Angelo V., Indolfi C., Indolfi P., Casale F. (2013). The Bcl-2/Bax and Ras/Raf/MEK/ERK signaling pathways: Implications in pediatric leukemia pathogenesis and new prospects for therapeutic approaches. Expert Rev. Hematol..

[16] Sakamoto K.M., Grant S., Saleiro D., Crispino J.D., Hijiya N., Giles F., Platanias L., Eklund E.A. (2015). Targeting novel signaling pathways for resistant acute myeloid leukemia. Mol. Genet. Metab..

[17] Reed J.C., Meister L., Tanaka S., Cuddy M., Yum S., Geyer C., Pleasure D. (1991). Differential expression of bcl2 protooncogene in neuroblastoma and other human tumor cell lines of neural origin. Cancer Res..

[18] Kuwashima Y., Uehara T., Kishi K., Shiromizu K., Matsuzawa M., Takayama S. (1994). Immunohistochemical characterization of undifferentiated carcinomas of the ovary. J. Cancer Res. Clin. Oncol..

[19] Monaghan P., Robertson D., Amos T.A., Dyer M.J., Mason D.Y., Greaves M.F. (1992). Ultrastructural localization of bcl-2 protein. J. Histochem. Cytochem..

[20] Pezzella F., Turley H., Kuzu I., Tungekar M.F., Dunnill M.S., Pierce C.B., Harris A., Gatter K.C., Mason D.Y. (1993). Bcl-2 Protein in Non-Small-Cell Lung Carcinoma. N. Engl. J. Med..

[21] Hartman M.L., Czyz M. (2013). Anti-apoptotic proteins on guard of melanoma cell survival. Cancer Lett..

[22] Mukherjee N., Schwan J.V., Fujita M., Norris D.A., Shellman Y.G. (2015). Alternative Treatments For Melanoma: Targeting BCL-2 Family Members to De-Bulk and Kill Cancer Stem Cells. J. Investig. Dermatol..

[23] Geng M., Wang L., Li P. (2013). Correlation between chemosensitivity to anticancer drugs and Bcl-2 expression in gastric cancer. Int. J. Clin. Exp. Pathol..

[24] Yang X.K., Zheng F., Chen J.H., Gao Q.L., Lu Y.P., Wang S.X., Wang C.Y., Ma D. (2002). Relationship between expression of apoptosis-associated proteins and caspase-3 activity in cisplatin-resistant human ovarian cancer cell line. Ai Zheng.

[25] Zhao Y., Zhang C.L., Zeng B.F., Wu X.S., Gao T.T., Oda Y. (2009). Enhanced chemosensitivity of drug-resistant osteosarcoma cells by lentivirus-mediated Bcl-2 silencing. Biochem. Biophys. Res. Commun..

[26] Kim D.W., Kim K.O., Shin M.J., Ha J.H., Seo S.W., Yang J., Lee F.Y. (2009). siRNA-based targeting of antiapoptotic genes can reverse chemoresistance in P-glycoprotein expressing chondrosarcoma cells. Mol. Cancer.

[27] Maji S., Panda S., Samal S.K., Shriwas O., Rath R., Pellecchia M., Emdad L., Das S.K., Fisher P.B., Dash R. (2018). Bcl-2 Antiapoptotic Family Proteins and Chemoresistance in Cancer. Adv. Cancer Res..

[28] Trisciuoglio D., Desideri M., Ciuffreda L., Mottolese M., Ribatti D., Vacca A., Del Rosso M., Marcocci L., Zupi G., Del Bufalo D. (2005). Bcl-2 overexpression in melanoma cells increases tumor progression-associated properties and in vivo tumor growth. J. Cell. Physiol..

[29] Trisciuoglio D., Iervolino A., Zupi G., Del Bufalo D. (2005). Involvement of PI3K and MAPK signaling in bcl-2-induced vascular endothelial growth factor expression in melanoma cells. Mol. Biol. Cell.

[30] Trisciuoglio D., Gabellini C., Desideri M., Ziparo E., Zupi G., Del Bufalo D. (2010). Bcl-2 regulates HIF-1alpha protein stabilization in hypoxic melanoma cells via the molecular chaperone HSP90. PLoS ONE.

[31] Trisciuoglio D., Gabellini C., Desideri M., Ragazzoni Y., De Luca T., Ziparo E., Del Bufalo D. (2011). Involvement of BH4 domain of bcl-2 in the regulation of HIF-1-mediated VEGF expression in hypoxic tumor cells. Cell Death Differ..

[32] Trisciuoglio D., De Luca T., Desideri M., Passeri D., Gabellini C., Scarpino S., Liang C., Orlandi A., Del Bufalo D. (2013). Removal of the BH4 domain from Bcl-2 protein triggers an autophagic process that impairs tumor growth. Neoplasia.

[33] Gabellini C., De Luca T., Trisciuoglio D., Desideri M., Di Martile M., Passeri D., Candiloro A., Biffoni M., Rizzo M.G., Orlandi A. (2013). BH4 domain of bcl-2 protein is required for its proangiogenic function under hypoxic condition. Carcinogenesis.

[34] Trisciuoglio D., Tupone M.G., Desideri M., Di Martile M., Gabellini C., Buglioni S., Pallocca M., Alessandrini G., D’Aguanno S., Del Bufalo D. (2017). BCL-XL overexpression promotes tumor progression-associated properties. Cell Death Dis..

[35] De Luca T., Pelosi A., Trisciuoglio D., D’Aguanno S., Desideri M., Farini V., Di Martile M., Bellei B., Tupone M.G., Candiloro A. (2016). miR-211 and MITF modulation by Bcl-2 protein in melanoma cells. Mol. Carcinog..

[36] D’Aguanno S., Valentini E., Tupone M.G., Desideri M., Di Martile M., Spagnuolo M., Buglioni S., Ercolani C., Falcone I., De Dominici M. (2018). Semaphorin 5A drives melanoma progression: Role of Bcl-2, miR-204 and c-Myb. J. Exp. Clin. Cancer Res..

[37] Tupone M.G., D’Aguanno S., Di Martile M., Valentini E., Desideri M., Trisciuoglio D., Donzelli S., Sacconi A., Buglioni S., Ercolani C. (2020). microRNA-378a-5p iS a novel positive regulator of melanoma progression. Oncogenesis.

[38] Di Martile M., Farini V., Consonni F.M., Trisciuoglio D., Desideri M., Valentini E., D’Aguanno S., Tupone M.G., Buglioni S., Ercolani C. (2020). Melanoma-specific bcl-2 promotes a protumoral M2-like phenotype by tumor-associated macrophages. J. Immunother. Cancer.

[39] Trisciuoglio D., Desideri M., Farini V., De Luca T., Di Martile M., Tupone M.G., Urbani A., D’Aguanno S., Del Bufalo D. (2016). Affinity purification-mass spectrometry analysis of bcl-2 interactome identified SLIRP as a novel interacting protein. Cell. Death Dis..

[40] Chong S.J.F., Marchi S., Petroni G., Kroemer G., Galluzzi L., Pervaiz S. (2020). Noncanonical Cell Fate Regulation by Bcl-2 Proteins. Trends Cell Biol..

[41] Oliver L., Cordel S., Barbieux I., LeCabellec M.T., Meflah K., Gregoire M., Vallette F.M. (2002). Resistance to apoptosis is increased during metastatic dissemination of colon cancer. Clin. Exp. Metastasis.

[42] Melucci E., Cosimelli M., Carpanese L., Pizzi G., Izzo F., Fiore F., Golfieri R., Giampalma E., Sperduti I., Ercolani C. (2013). Italian Society of Locoregional Therapies in Oncology (S.I.T.I.L.O.) Decrease of survivin, p53 and Bcl-2 expression in chemorefractory colorectal liver metastases may be predictive of radiosensivity radiosensivity after radioembolization with yttrium-90 resin microspheres. J. Exp. Clin. Cancer Res..

[43] Neri A., Marrelli D., Roviello F., DeMarco G., Mariani F., DeStefano A., Megha T., Caruso S., Corso G., Cioppa T. (2006). Bcl-2 expression correlates with lymphovascular invasion and long-term prognosis in breast cancer. Breast Cancer Res. Treat..

[44] Tawfik K., Kimler B.F., Davis M.K., Fan F., Tawfik O. (2012). Prognostic significance of Bcl-2 in invasive mammary carcinomas: A comparative clinicopathologic study between “triple-negative” and non-”triple-negative” tumors. Hum. Pathol..

[45] Gryko M., Pryczynicz A., Zareba K., Kedra B., Kemona A., Guzinska-Ustymowicz K. (2014). The expression of Bcl-2 and BID in gastric cancer cells. J. Immunol. Res..

[46] Groeger A.M., Caputi M., Esposito V., De Luca A., Salat A., Murabito M., Giordano G.G., Baldi F., Giordano A., Wolner E. (1999). Bcl-2 protein expression correlates with nodal status in non small cell lung cancer. Anticancer Res..

[47] Stevens M., Oltean S. (2019). Modulation of the Apoptosis Gene Bcl-x Function Through Alternative Splicing. Front. Genet..

[48] Warren C.F.A., Wong-Brown M.W., Bowden N.A. (2019). BCL-2 family isoforms in apoptosis and cancer. Cell. Death Dis..

[49] Giorgini S., Trisciuoglio D., Gabellini C., Desideri M., Castellini L., Colarossi C., Zangemeister-Wittke U., Zupi G., Del Bufalo D. (2007). Modulation of bcl-xL in tumor cells regulates angiogenesis through CXCL8 expression. Mol. Cancer Res..

[50] Gabellini C., Castellini L., Trisciuoglio D., Kracht M., Zupi G., Del Bufalo D. (2008). Involvement of nuclear factor-kappa B in bcl-xL-induced interleukin 8 expression in glioblastoma. J. Neurochem..

[51] Gabellini C., Gomez-Abenza E., Ibanez-Molero S., Tupone M.G., Perez-Oliva A.B., de Oliveira S., Del Bufalo D., Mulero V. (2018). Interleukin 8 mediates bcl-xL-induced enhancement of human melanoma cell dissemination and angiogenesis in a zebrafish xenograft model. Int. J. Cancer.

[52] Choi S., Chen Z., Tang L.H., Fang Y., Shin S.J., Panarelli N.C., Chen Y.T., Li Y., Jiang X., Du Y.N. (2016). Bcl-xL promotes metastasis independent of its anti-apoptotic activity. Nat. Commun..

[53] Bessou M., Lopez J., Gadet R., Deygas M., Popgeorgiev N., Poncet D., Nougarede A., Billard P., Mikaelian I., Gonzalo P. (2020). The apoptosis inhibitor Bcl-xL controls breast cancer cell migration through mitochondria-dependent reactive oxygen species production. Oncogene.

[54] Singh L., Pushker N., Saini N., Sen S., Sharma A., Bakhshi S., Chawla B., Kashyap S. (2015). Expression of pro-apoptotic Bax and anti-apoptotic Bcl-2 proteins in human retinoblastoma. Clin. Exp. Ophthalmol..

[55] Zhang H., Rosdahl I. (2006). Bcl-xL and bcl-2 proteins in melanoma progression and UVB-induced apoptosis. Int. J. Oncol..

[56] Keitel U., Scheel A., Thomale J., Halpape R., Kaulfuss S., Scheel C., Dobbelstein M. (2014). Bcl-xL mediates therapeutic resistance of a mesenchymal breast cancer cell subpopulation. Oncotarget.

[57] Zhang Y.L., Pang L.Q., Wu Y., Wang X.Y., Wang C.Q., Fan Y. (2008). Significance of Bcl-xL in human colon carcinoma. World J. Gastroenterol..

[58] Zhang K., Jiao K., Xing Z., Zhang L., Yang J., Xie X., Yang L. (2014). Bcl-xL overexpression and its association with the progress of tongue carcinoma. Int. J. Clin. Exp. Pathol..

[59] Watanabe J., Kushihata F., Honda K., Sugita A., Tateishi N., Mominoki K., Matsuda S., Kobayashi N. (2004). Prognostic significance of Bcl-xL in human hepatocellular carcinoma. Surgery.

[60] Kozopas K.M., Yang T., Buchan H.L., Zhou P., Craig R.W. (1993). MCL1, a gene expressed in programmed myeloid cell differentiation, has sequence similarity to BCL2. Proc. Natl. Acad. Sci. USA.

[61] Beroukhim R., Mermel C.H., Porter D., Wei G., Raychaudhuri S., Donovan J., Barretina J., Boehm J.S., Dobson J., Urashima M. (2010). The landscape of somatic copy-number alteration across human cancers. Nature.

[62] Campbell K.J., Dhayade S., Ferrari N., Sims A.H., Johnson E., Mason S.M., Dickson A., Ryan K.M., Kalna G., Edwards J. (2018). MCL-1 is a prognostic indicator and drug target in breast cancer. Cell. Death Dis..

[63] Young A.I., Law A.M., Castillo L., Chong S., Cullen H.D., Koehler M., Herzog S., Brummer T., Lee E.F., Fairlie W.D. (2016). MCL-1 inhibition provides a new way to suppress breast cancer metastasis and increase sensitivity to dasatinib. Breast Cancer Res..

[64] Zervantonakis I.K., Iavarone C., Chen H.Y., Selfors L.M., Palakurthi S., Liu J.F., Drapkin R., Matulonis U., Leverson J.D., Sampath D. (2017). Systems analysis of apoptotic priming in ovarian cancer identifies vulnerabilities and predictors of drug response. Nat. Commun..

[65] Reiner T., de Las Pozas A., Parrondo R., Palenzuela D., Cayuso W., Rai P., Perez-Stable C. (2015). Mcl-1 protects prostate cancer cells from cell death mediated by chemotherapy-induced DNA damage. Oncoscience.

[66] Akgul C. (2009). Mcl-1 is a potential therapeutic target in multiple types of cancer. Cell Mol. Life Sci..

[67] He K., Chen D., Ruan H., Li X., Tong J., Xu X., Zhang L., Yu J. (2016). BRAFV600E-dependent Mcl-1 stabilization leads to everolimus resistance in colon cancer cells. Oncotarget.

[68] Ma J., Zhao Z., Wu K., Xu Z., Liu K. (2016). MCL-1 is the key target of adjuvant chemotherapy to reverse the cisplatin-resistance in NSCLC. Gene.

[69] Awan F.T., Kay N.E., Davis M.E., Wu W., Geyer S.M., Leung N., Jelinek D.F., Tschumper R.C., Secreto C.R., Lin T.S. (2009). Mcl-1 expression predicts progression-free survival in chronic lymphocytic leukemia patients treated with pentostatin, cyclophosphamide, and rituximab. Blood.

[70] Lee W.S., Park Y.L., Kim N., Oh H.H., Son D.J., Kim M.Y., Oak C.Y., Chung C.Y., Park H.C., Kim J.S. (2015). Myeloid cell leukemia-1 regulates the cell growth and predicts prognosis in gastric cancer. Int. J. Oncol..

[71] Bashari M.H., Fan F., Vallet S., Sattler M., Arn M., Luckner-Minden C., Schulze-Bergkamen H., Zornig I., Marme F., Schneeweiss A. (2016). Mcl-1 confers protection of Her2-positive breast cancer cells to hypoxia: Therapeutic implications. Breast Cancer Res..

[72] Wen Q., Zhan Y., Zheng H., Zang H., Luo J., Zhang Y., Wang W., Feng J., Lu J., Chen L. (2019). Elevated expression of mcl-1 inhibits apoptosis and predicts poor prognosis in patients with surgically resected non-small cell lung cancer. Diagn. Pathol..

[73] Del Bufalo D., Rizzo A., Trisciuoglio D., Cardinali G., Torrisi M.R., Zangemeister-Wittke U., Zupi G., Biroccio A. (2005). Involvement of hTERT in apoptosis induced by interference with Bcl-2 expression and function. Cell Death Differ..

[74] Zangemeister-Wittke U., Leech S.H., Olie R.A., Simoes-Wust A.P., Gautschi O., Luedke G.H., Natt F., Haner R., Martin P., Hall J. (2000). A novel bispecific antisense oligonucleotide inhibiting both bcl-2 and bcl-xL expression efficiently induces apoptosis in tumor cells. Clin. Cancer Res..

[75] Olie R.A., Hafner C., Kuttel R., Sigrist B., Willers J., Dummer R., Hall J., Stahel R.A., Zangemeister-Wittke U. (2002). Bcl-2 and bcl-xL antisense oligonucleotides induce apoptosis in melanoma cells of different clinical stages. J. Investig. Dermatol..

[76] Bedikian A.Y., Garbe C., Conry R., Lebbe C., Grob J.J. (2014). Genasense Melanoma Study Group Dacarbazine with or without oblimersen (a Bcl-2 antisense oligonucleotide) in chemotherapy-naive patients with advanced melanoma and low-normal serum lactate dehydrogenase: ‘The AGENDA trial’. Melanoma Res..

[77] Raab R., Sparano J.A., Ocean A.J., Christos P., Ramirez M., Vinciguerra V., Kaubisch A. (2010). A phase I trial of oblimersen sodium in combination with cisplatin and 5-fluorouracil in patients with advanced esophageal, gastroesophageal junction, and gastric carcinoma. Am. J. Clin. Oncol..

[78] Galatin P.S., Advani R.H., Fisher G.A., Francisco B., Julian T., Losa R., Sierra M.I., Sikic B.I. (2011). Phase I trial of oblimersen (Genasense(R)) and gemcitabine in refractory and advanced malignancies. Investig. New Drugs.

[79] Ott P.A., Chang J., Madden K., Kannan R., Muren C., Escano C., Cheng X., Shao Y., Mendoza S., Gandhi A. (2013). Oblimersen in combination with temozolomide and albumin-bound paclitaxel in patients with advanced melanoma: A phase I trial. Cancer Chemother. Pharmacol..

[80] Leverson J.D., Sampath D., Souers A.J., Rosenberg S.H., Fairbrother W.J., Amiot M., Konopleva M., Letai A. (2017). Found in Translation: How Preclinical Research Is Guiding the Clinical Development of the BCL2-Selective Inhibitor Venetoclax. Cancer Discov..

[81] Delbridge A.R., Grabow S., Strasser A., Vaux D.L. (2016). Thirty years of BCL-2: Translating cell death discoveries into novel cancer therapies. Nat. Rev. Cancer.

[82] Merino D., Kelly G.L., Lessene G., Wei A.H., Roberts A.W., Strasser A. (2018). BH3-Mimetic Drugs: Blazing the Trail for New Cancer Medicines. Cancer Cell.

[83] Roberts A.W., Seymour J.F., Brown J.R., Wierda W.G., Kipps T.J., Khaw S.L., Carney D.A., He S.Z., Huang D.C., Xiong H. (2012). Substantial susceptibility of chronic lymphocytic leukemia to BCL2 inhibition: Results of a phase I study of navitoclax in patients with relapsed or refractory disease. J. Clin. Oncol..

[84] Inoue-Yamauchi A., Jeng P.S., Kim K., Chen H.C., Han S., Ganesan Y.T., Ishizawa K., Jebiwott S., Dong Y., Pietanza M.C. (2017). Targeting the differential addiction to anti-apoptotic BCL-2 family for cancer therapy. Nat. Commun..

[85] Aubrey B.J., Kelly G.L., Kueh A.J., Brennan M.S., O’Connor L., Milla L., Wilcox S., Tai L., Strasser A., Herold M.J. (2015). An inducible lentiviral guide RNA platform enables the identification of tumor-essential genes and tumor-promoting mutations in vivo. Cell. Rep..

[86] Kotschy A., Szlavik Z., Murray J., Davidson J., Maragno A.L., Le Toumelin-Braizat G., Chanrion M., Kelly G.L., Gong J.N., Moujalled D.M. (2016). The MCL1 inhibitor S63845 is tolerable and effective in diverse cancer models. Nature.

[87] Zhai D., Jin C., Satterthwait A.C., Reed J.C. (2006). Comparison of chemical inhibitors of antiapoptotic Bcl-2-family proteins. Cell Death Differ..

[88] Paulus A., Chitta K., Akhtar S., Personett D., Miller K.C., Thompson K.J., Carr J., Kumar S., Roy V., Ansell S.M. (2014). AT-101 downregulates BCL2 and MCL1 and potentiates the cytotoxic effects of lenalidomide and dexamethasone in preclinical models of multiple myeloma and Waldenstrom macroglobulinaemia. Br. J. Haematol..

[89] Oliver C.L., Miranda M.B., Shangary S., Land S., Wang S., Johnson D.E. (2005). (-)-Gossypol acts directly on the mitochondria to overcome Bcl-2- and Bcl-X(L)-mediated apoptosis resistance. Mol. Cancer Ther..

[90] Nguyen M., Marcellus R.C., Roulston A., Watson M., Serfass L., Murthy Madiraju S.R., Goulet D., Viallet J., Belec L., Billot X. (2007). Small molecule obatoclax (GX15-070) antagonizes MCL-1 and overcomes MCL-1-mediated resistance to apoptosis. Proc. Natl. Acad. Sci. USA.

[91] Hu Y., Yague E., Zhao J., Wang L., Bai J., Yang Q., Pan T., Zhao H., Liu J., Zhang J. (2018). Sabutoclax, pan-active BCL-2 protein family antagonist, overcomes drug resistance and eliminates cancer stem cells in breast cancer. Cancer Lett..

[92] Quinn B.A., Dash R., Sarkar S., Azab B., Bhoopathi P., Das S.K., Emdad L., Wei J., Pellecchia M., Sarkar D. (2015). Pancreatic Cancer Combination Therapy Using a BH3 Mimetic and a Synthetic Tetracycline. Cancer Res..

[93] Jackson R.S., Placzek W., Fernandez A., Ziaee S., Chu C.Y., Wei J., Stebbins J., Kitada S., Fritz G., Reed J.C. (2012). Sabutoclax, a Mcl-1 antagonist, inhibits tumorigenesis in transgenic mouse and human xenograft models of prostate cancer. Neoplasia.

[94] Oltersdorf T., Elmore S.W., Shoemaker A.R., Armstrong R.C., Augeri D.J., Belli B.A., Bruncko M., Deckwerth T.L., Dinges J., Hajduk P.J. (2005). An inhibitor of Bcl-2 family proteins induces regression of solid tumours. Nature.

[95] Del Gaizo Moore V., Brown J.R., Certo M., Love T.M., Novina C.D., Letai A. (2007). Chronic lymphocytic leukemia requires BCL2 to sequester prodeath BIM, explaining sensitivity to BCL2 antagonist ABT-737. J. Clin. Investig..

[96] Sakakibara-Konishi J., Ikezawa Y., Oizumi S., Kikuchi J., Kikuchi E., Mizugaki H., Kinoshita I., Dosaka-Akita H., Nishimura M. (2017). Combined antitumor effect of gamma-secretase inhibitor and ABT-737 in Notch-expressing non-small cell lung cancer. Int. J. Clin. Oncol..

[97] Yu X., Dobrikov M., Keir S.T., Gromeier M., Pastan I.H., Reisfeld R., Bigner D.D., Chandramohan V. (2019). Synergistic antitumor effects of 9.2.27-PE38KDEL and ABT-737 in primary and metastatic brain tumors. PLoS ONE.

[98] Serasinghe M.N., Missert D.J., Asciolla J.J., Podgrabinska S., Wieder S.Y., Izadmehr S., Belbin G., Skobe M., Chipuk J.E. (2015). Anti-apoptotic BCL-2 proteins govern cellular outcome following B-RAF(V600E) inhibition and can be targeted to reduce resistance. Oncogene.

[99] Suryani S., Carol H., Chonghaile T.N., Frismantas V., Sarmah C., High L., Bornhauser B., Cowley M.J., Szymanska B., Evans K. (2014). Cell and molecular determinants of in vivo efficacy of the BH3 mimetic ABT-263 against pediatric acute lymphoblastic leukemia xenografts. Clin. Cancer Res..

[100] Faber A.C., Farago A.F., Costa C., Dastur A., Gomez-Caraballo M., Robbins R., Wagner B.L., Rideout W.M., Jakubik C.T., Ham J. (2015). Assessment of ABT-263 activity across a cancer cell line collection leads to a potent combination therapy for small-cell lung cancer. Proc. Natl. Acad. Sci. USA.

[101] Schulze A.B., Evers G., Kerkhoff A., Mohr M., Schliemann C., Berdel W.E., Schmidt L.H. (2019). Future Options of Molecular-Targeted Therapy in Small Cell Lung Cancer. Cancers (Basel).

[102] Kim Y.J., Tsang T., Anderson G.R., Posimo J.M., Brady D.C. (2020). Inhibition of BCL2 Family Members Increases the Efficacy of Copper Chelation in BRAF(V600E)-Driven Melanoma. Cancer Res..

[103] Schoenwaelder S.M., Jarman K.E., Gardiner E.E., Hua M., Qiao J., White M.J., Josefsson E.C., Alwis I., Ono A., Willcox A. (2011). Bcl-xL-inhibitory BH3 mimetics can induce a transient thrombocytopathy that undermines the hemostatic function of platelets. Blood.

[104] Khan S., Zhang X., Lv D., Zhang Q., He Y., Zhang P., Liu X., Thummuri D., Yuan Y., Wiegand J.S. (2019). A selective BCL-XL PROTAC degrader achieves safe and potent antitumor activity. Nat. Med..

[105] Wilson W.H., O’Connor O.A., Czuczman M.S., LaCasce A.S., Gerecitano J.F., Leonard J.P., Tulpule A., Dunleavy K., Xiong H., Chiu Y.L. (2010). Navitoclax, a targeted high-affinity inhibitor of BCL-2, in lymphoid malignancies: A phase 1 dose-escalation study of safety, pharmacokinetics, pharmacodynamics, and antitumour activity. Lancet Oncol..

[106] Inao T., Iida Y., Moritani T., Okimoto T., Tanino R., Kotani H., Harada M. (2018). Bcl-2 inhibition sensitizes triple-negative human breast cancer cells to doxorubicin. Oncotarget.

[107] Wali V.B., Langdon C.G., Held M.A., Platt J.T., Patwardhan G.A., Safonov A., Aktas B., Pusztai L., Stern D.F., Hatzis C. (2017). Systematic Drug Screening Identifies Tractable Targeted Combination Therapies in Triple-Negative Breast Cancer. Cancer Res..

[108] Fleury H., Malaquin N., Tu V., Gilbert S., Martinez A., Olivier M.A., Sauriol A., Communal L., Leclerc-Desaulniers K., Carmona E. (2019). Exploiting interconnected synthetic lethal interactions between PARP inhibition and cancer cell reversible senescence. Nat. Commun..

[109] Faber A.C., Coffee E.M., Costa C., Dastur A., Ebi H., Hata A.N., Yeo A.T., Edelman E.J., Song Y., Tam A.T. (2014). mTOR inhibition specifically sensitizes colorectal cancers with KRAS or BRAF mutations to BCL-2/BCL-XL inhibition by suppressing MCL-1. Cancer. Discov..

[110] Bai L., Chen J., McEachern D., Liu L., Zhou H., Aguilar A., Wang S. (2014). BM-1197: A novel and specific Bcl-2/Bcl-xL inhibitor inducing complete and long-lasting tumor regression in vivo. PLoS ONE.

[111] Ye L., Yuan G., Xu F., Sun Y., Chen Z., Chen M., Li T., Sun P., Li S., Sun J. (2015). The small-molecule compound BM-1197 inhibits the antiapoptotic regulators Bcl-2/Bcl-xL and triggers apoptotic cell death in human colorectal cancer cells. Tumour Biol..

[112] Sun Y.L., Jiang W.Q., Luo Q.Y., Yang D.J., Cai Y.C., Huang H.Q., Sun J. (2019). A novel Bcl-2 inhibitor, BM-1197, induces apoptosis in malignant lymphoma cells through the endogenous apoptotic pathway. BMC Cancer.

[113] Loriot Y., Mordant P., Dugue D., Geneste O., Gombos A., Opolon P., Guegan J., Perfettini J.L., Pierre A., Berthier L.K. (2014). Radiosensitization by a novel Bcl-2 and Bcl-XL inhibitor S44563 in small-cell lung cancer. Cell Death Dis..

[114] Nemati F., de Montrion C., Lang G., Kraus-Berthier L., Carita G., Sastre-Garau X., Berniard A., Vallerand D., Geneste O., de Plater L. (2014). Targeting Bcl-2/Bcl-XL induces antitumor activity in uveal melanoma patient-derived xenografts. PLoS ONE.

[115] Wang J., Yang D., Luo Q., Qiu M., Zhang L., Li B., Chen H., Yi H., Yan X., Li S. (2017). APG-1252-12A induces mitochondria-dependent apoptosis through inhibiting the antiapoptotic proteins Bcl-2/Bcl-xl in HL-60 cells. Int. J. Oncol..

[116] Zhou H., Aguilar A., Chen J., Bai L., Liu L., Meagher J.L., Yang C.Y., McEachern D., Cong X., Stuckey J.A. (2012). Structure-based design of potent Bcl-2/Bcl-xL inhibitors with strong in vivo antitumor activity. J. Med. Chem..

[117] Zhu J., Wang Z., Guo Z., Zhang X., Song T., Guo Y., Ji T., Zhang Z. (2020). Structure-based design, synthesis, and evaluation of Bcl-2/Mcl-1 dual inhibitors. Arch. Pharm. (Weinheim).

[118] Abou Samra A., Robert A., Gov C., Favre L., Eloy L., Jacquet E., Bignon J., Wiels J., Desrat S., Roussi F. (2018). Dual inhibitors of the pro-survival proteins Bcl-2 and Mcl-1 derived from natural compound meiogynin A. Eur. J. Med. Chem..

[119] Desrat S., Pujals A., Colas C., Dardenne J., Geny C., Favre L., Dumontet V., Iorga B.I., Litaudon M., Raphael M. (2014). Pro-apoptotic meiogynin A derivatives that target Bcl-xL and Mcl-1. Bioorg. Med. Chem. Lett..

[120] Wang Z., He N., Guo Z., Niu C., Song T., Guo Y., Cao K., Wang A., Zhu J., Zhang X. (2019). Proteolysis Targeting Chimeras for the Selective Degradation of Mcl-1/Bcl-2 Derived from Nonselective Target Binding Ligands. J. Med. Chem..

[121] Lee E.F., Harris T.J., Tran S., Evangelista M., Arulananda S., John T., Ramnac C., Hobbs C., Zhu H., Gunasingh G. (2019). BCL-XL and MCL-1 are the key BCL-2 family proteins in melanoma cell survival. Cell Death Dis..

[122] Souers A.J., Leverson J.D., Boghaert E.R., Ackler S.L., Catron N.D., Chen J., Dayton B.D., Ding H., Enschede S.H., Fairbrother W.J. (2013). ABT-199, a potent and selective BCL-2 inhibitor, achieves antitumor activity while sparing platelets. Nat. Med..

[123] Roberts A.W., Davids M.S., Pagel J.M., Kahl B.S., Puvvada S.D., Gerecitano J.F., Kipps T.J., Anderson M.A., Brown J.R., Gressick L. (2016). Targeting BCL2 with Venetoclax in Relapsed Chronic Lymphocytic Leukemia. N. Engl. J. Med..

[124] Lachowiez C., DiNardo C.D., Konopleva M. (2020). Venetoclax in acute myeloid leukemia-current and future directions. Leuk. Lymphoma.

[125] Seymour J.F., Kipps T.J., Eichhorst B., Hillmen P., D’Rozario J., Assouline S., Owen C., Gerecitano J., Robak T., De la Serna J. (2018). Venetoclax-Rituximab in Relapsed or Refractory Chronic Lymphocytic Leukemia. N. Engl. J. Med..

[126] Flinn I.W., Gribben J.G., Dyer M.J.S., Wierda W., Maris M.B., Furman R.R., Hillmen P., Rogers K.A., Iyer S.P., Quillet-Mary A. (2019). Phase 1b study of venetoclax-obinutuzumab in previously untreated and relapsed/refractory chronic lymphocytic leukemia. Blood.

[127] Jain N., Keating M., Thompson P., Ferrajoli A., Burger J., Borthakur G., Takahashi K., Estrov Z., Fowler N., Kadia T. (2019). Ibrutinib and Venetoclax for First-Line Treatment of CLL. N. Engl. J. Med..

[128] Kumar S., Kaufman J.L., Gasparetto C., Mikhael J., Vij R., Pegourie B., Benboubker L., Facon T., Amiot M., Moreau P. (2017). Efficacy of venetoclax as targeted therapy for relapsed/refractory t(11;14) multiple myeloma. Blood.

[129] Moreau P., Chanan-Khan A., Roberts A.W., Agarwal A.B., Facon T., Kumar S., Touzeau C., Punnoose E.A., Cordero J., Munasinghe W. (2017). Promising efficacy and acceptable safety of venetoclax plus bortezomib and dexamethasone in relapsed/refractory MM. Blood.

[130] Konopleva M., Pollyea D.A., Potluri J., Chyla B., Hogdal L., Busman T., McKeegan E., Salem A.H., Zhu M., Ricker J.L. (2016). Efficacy and Biological Correlates of Response in a Phase II Study of Venetoclax Monotherapy in Patients with Acute Myelogenous Leukemia. Cancer. Discov..

[131] Chan S.M., Thomas D., Corces-Zimmerman M.R., Xavy S., Rastogi S., Hong W.J., Zhao F., Medeiros B.C., Tyvoll D.A., Majeti R. (2015). Isocitrate dehydrogenase 1 and 2 mutations induce BCL-2 dependence in acute myeloid leukemia. Nat. Med..

[132] Birkinshaw R.W., Gong J.N., Luo C.S., Lio D., White C.A., Anderson M.A., Blombery P., Lessene G., Majewski I.J., Thijssen R. (2019). Structures of BCL-2 in complex with venetoclax reveal the molecular basis of resistance mutations. Nat. Commun..

[133] Lok S.W., Whittle J.R., Vaillant F., Teh C.E., Lo L.L., Policheni A.N., Bergin A.R.T., Desai J., Ftouni S., Gandolfo L.C. (2019). A Phase Ib Dose-Escalation and Expansion Study of the BCL2 Inhibitor Venetoclax Combined with Tamoxifen in ER and BCL2-Positive Metastatic Breast Cancer. Cancer Discov..

[134] Casara P., Davidson J., Claperon A., Le Toumelin-Braizat G., Vogler M., Bruno A., Chanrion M., Lysiak-Auvity G., Le Diguarher T., Starck J.B. (2018). S55746 is a novel orally active BCL-2 selective and potent inhibitor that impairs hematological tumor growth. Oncotarget.

[135] Karpel-Massler G., Ishida C.T., Bianchetti E., Shu C., Perez-Lorenzo R., Horst B., Banu M., Roth K.A., Bruce J.N., Canoll P. (2017). Inhibition of Mitochondrial Matrix Chaperones and Antiapoptotic Bcl-2 Family Proteins Empower Antitumor Therapeutic Responses. Cancer Res..

[136] Whittle J.R., Vaillant F., Surgenor E., Policheni A.N., Giner G., Capaldo B.D., Chen H.R., Liu H.K., Dekkers J.F., Sachs N. (2020). Dual targeting of CDK4/6 and BCL2 pathways augments tumor response in estrogen receptor positive breast cancer. Clin. Cancer Res..

[137] Liu W., Krump N.A., Herlyn M., You J. (2020). Combining DNA Damage Induction with BCL-2 Inhibition to Enhance Merkel Cell Carcinoma Cytotoxicity. Biology (Basel).

[138] Tahir S.K., Smith M.L., Hessler P., Rapp L.R., Idler K.B., Park C.H., Leverson J.D., Lam L.T. (2017). Potential mechanisms of resistance to venetoclax and strategies to circumvent it. BMC Cancer.

[139] Leverson J.D., Phillips D.C., Mitten M.J., Boghaert E.R., Diaz D., Tahir S.K., Belmont L.D., Nimmer P., Xiao Y., Ma X.M. (2015). Exploiting selective BCL-2 family inhibitors to dissect cell survival dependencies and define improved strategies for cancer therapy. Sci. Transl. Med..

[140] Bose P., Gandhi V., Konopleva M. (2017). Pathways and mechanisms of venetoclax resistance. Leuk. Lymphoma.

[141] Lessene G., Czabotar P.E., Sleebs B.E., Zobel K., Lowes K.N., Adams J.M., Baell J.B., Colman P.M., Deshayes K., Fairbrother W.J. (2013). Structure-guided design of a selective BCL-X(L) inhibitor. Nat. Chem. Biol..

[142] Baranski Z., de Jong Y., Ilkova T., Peterse E.F., Cleton-Jansen A.M., van de Water B., Hogendoorn P.C., Bovee J.V., Danen E.H. (2015). Pharmacological inhibition of Bcl-xL sensitizes osteosarcoma to doxorubicin. Oncotarget.

[143] Tao Z.F., Hasvold L., Wang L., Wang X., Petros A.M., Park C.H., Boghaert E.R., Catron N.D., Chen J., Colman P.M. (2014). Discovery of a Potent and Selective BCL-XL Inhibitor with in Vivo Activity. ACS Med. Chem. Lett..

[144] Faqar-Uz-Zaman S.F., Heinicke U., Meister M.T., Vogler M., Fulda S. (2018). BCL-xL-selective BH3 mimetic sensitizes rhabdomyosarcoma cells to chemotherapeutics by activation of the mitochondrial pathway of apoptosis. Cancer Lett..

[145] Wei A.H., Roberts A.W., Spencer A., Rosenberg A.S., Siegel D., Walter R.B., Caenepeel S., Hughes P., McIver Z., Mezzi K. (2020). Targeting MCL-1 in hematologic malignancies: Rationale and progress. Blood Rev..

[146] Abulwerdi F., Liao C., Liu M., Azmi A.S., Aboukameel A., Mady A.S., Gulappa T., Cierpicki T., Owens S., Zhang T. (2014). A novel small-molecule inhibitor of mcl-1 blocks pancreatic cancer growth in vitro and in vivo. Mol. Cancer Ther..

[147] Leverson J.D., Zhang H., Chen J., Tahir S.K., Phillips D.C., Xue J., Nimmer P., Jin S., Smith M., Xiao Y. (2015). Potent and selective small-molecule MCL-1 inhibitors demonstrate on-target cancer cell killing activity as single agents and in combination with ABT-263 (navitoclax). Cell Death Dis..

[148] Xiao Y., Nimmer P., Sheppard G.S., Bruncko M., Hessler P., Lu X., Roberts-Rapp L., Pappano W.N., Elmore S.W., Souers A.J. (2015). MCL-1 Is a Key Determinant of Breast Cancer Cell Survival: Validation of MCL-1 Dependency Utilizing a Highly Selective Small Molecule Inhibitor. Mol. Cancer Ther..

[149] Lee T., Bian Z., Zhao B., Hogdal L.J., Sensintaffar J.L., Goodwin C.M., Belmar J., Shaw S., Tarr J.C., Veerasamy N. (2017). Discovery and biological characterization of potent myeloid cell leukemia-1 inhibitors. FEBS Lett..

[150] Nangia V., Siddiqui F.M., Caenepeel S., Timonina D., Bilton S.J., Phan N., Gomez-Caraballo M., Archibald H.L., Li C., Fraser C. (2018). Exploiting MCL1 Dependency with Combination MEK + MCL1 Inhibitors Leads to Induction of Apoptosis and Tumor Regression in KRAS-Mutant Non-Small Cell Lung Cancer. Cancer Discov..

[151] Weeden C.E., Ah-Cann C., Holik A.Z., Pasquet J., Garnier J.M., Merino D., Lessene G., Asselin-Labat M.L. (2018). Dual inhibition of BCL-XL and MCL-1 is required to induce tumour regression in lung squamous cell carcinomas sensitive to FGFR inhibition. Oncogene.

[152] Karpel-Massler G., Ishida C.T., Zhang Y., Halatsch M.E., Westhoff M.A., Siegelin M.D. (2017). Targeting intrinsic apoptosis and other forms of cell death by BH3-mimetics in glioblastoma. Expert Opin. Drug Discov..

[153] Merino D., Whittle J.R., Vaillant F., Serrano A., Gong J.N., Giner G., Maragno A.L., Chanrion M., Schneider E., Pal B. (2017). Synergistic action of the MCL-1 inhibitor S63845 with current therapies in preclinical models of triple-negative and HER2-amplified breast cancer. Sci. Transl. Med..

[154] Abdul Rahman S.F., Muniandy K., Soo Y.K., Tiew E.Y.H., Tan K.X., Bates T.E., Mohana-Kumaran N. (2020). Co-inhibition of BCL-XL and MCL-1 with selective BCL-2 family inhibitors enhances cytotoxicity of cervical cancer cell lines. Biochem. Biophys. Rep..

[155] Kehr S., Haydn T., Bierbrauer A., Irmer B., Vogler M., Fulda S. (2020). Targeting BCL-2 proteins in pediatric cancer: Dual inhibition of BCL-XL and MCL-1 leads to rapid induction of intrinsic apoptosis. Cancer Lett..

[156] Tseng H.Y., Dreyer J., Emran A.A., Gunatilake D., Pirozyan M., Cullinane C., Dutton-Regester K., Rizos H., Hayward N.K., McArthur G. (2020). Co-targeting bromodomain and extra-terminal proteins and MCL1 induces synergistic cell death in melanoma. Int. J. Cancer.

[157] Thomas R.L., Roberts D.J., Kubli D.A., Lee Y., Quinsay M.N., Owens J.B., Fischer K.M., Sussman M.A., Miyamoto S., Gustafsson A.B. (2013). Loss of MCL-1 leads to impaired autophagy and rapid development of heart failure. Genes Dev..

[158] Wang X., Bathina M., Lynch J., Koss B., Calabrese C., Frase S., Schuetz J.D., Rehg J.E., Opferman J.T. (2013). Deletion of MCL-1 causes lethal cardiac failure and mitochondrial dysfunction. Genes Dev..

[159] Ramsey H.E., Fischer M.A., Lee T., Gorska A.E., Arrate M.P., Fuller L., Boyd K.L., Strickland S.A., Sensintaffar J., Hogdal L.J. (2018). A Novel MCL1 Inhibitor Combined with Venetoclax Rescues Venetoclax-Resistant Acute Myelogenous Leukemia. Cancer Discov..

[160] Williams M.M., Elion D.L., Rahman B., Hicks D.J., Sanchez V., Cook R.S. (2019). Therapeutic inhibition of Mcl-1 blocks cell survival in estrogen receptor-positive breast cancers. Oncotarget.

[161] Cidado J., Boiko S., Proia T., Ferguson D., Criscione S.W., San Martin M., Pop-Damkov P., Su N., Roamio Franklin V.N., Sekhar Reddy Chilamakuri C. (2020). AZD4573 Is a Highly Selective CDK9 Inhibitor That Suppresses MCL-1 and Induces Apoptosis in Hematologic Cancer Cells. Clin. Cancer Res..

[162] Berger S., Procko E., Margineantu D., Lee E.F., Shen B.W., Zelter A., Silva D.A., Chawla K., Herold M.J., Garnier J.M. (2016). Computationally designed high specificity inhibitors delineate the roles of BCL2 family proteins in cancer. eLife.

[163] Langan R.A., Boyken S.E., Ng A.H., Samson J.A., Dods G., Westbrook A.M., Nguyen T.H., Lajoie M.J., Chen Z., Berger S. (2019). De novo design of bioactive protein switches. Nature.

[164] Dutta S., Ryan J., Chen T.S., Kougentakis C., Letai A., Keating A.E. (2015). Potent and specific peptide inhibitors of human pro-survival protein Bcl-xL. J. Mol. Biol..

[165] Distelhorst C.W. (2018). Targeting Bcl-2-IP3 receptor interaction to treat cancer: A novel approach inspired by nearly a century treating cancer with adrenal corticosteroid hormones. Biochim. Biophys. Acta Mol. Cell. Res..

[166] Liu Z., Wild C., Ding Y., Ye N., Chen H., Wold E.A., Zhou J. (2016). BH4 domain of Bcl-2 as a novel target for cancer therapy. Drug Discov. Today.

[167] Han B., Park D., Li R., Xie M., Owonikoko T.K., Zhang G., Sica G.L., Ding C., Zhou J., Magis A.T. (2015). Small-Molecule Bcl2 BH4 Antagonist for Lung Cancer Therapy. Cancer Cell.

[168] Deng J., Park D., Wang M., Nooka A., Deng Q., Matulis S., Kaufman J., Lonial S., Boise L.H., Galipeau J. (2016). BCL2-BH4 antagonist BDA-366 suppresses human myeloma growth. Oncotarget.

[169] Song T., Wang Z., Ji F., Feng Y., Fan Y., Chai G., Li X., Li Z., Zhang Z. (2016). Deactivation of Mcl-1 by Dual-Function Small-Molecule Inhibitors Targeting the Bcl-2 Homology 3 Domain and Facilitating Mcl-1 Ubiquitination. Angew. Chem. Int. Ed. Engl..

[170] Lee S., Wales T.E., Escudero S., Cohen D.T., Luccarelli J., Gallagher C.G., Cohen N.A., Huhn A.J., Bird G.H., Engen J.R. (2016). Allosteric inhibition of antiapoptotic MCL-1. Nat. Struct. Mol. Biol..

[171] Akcay G., Belmonte M.A., Aquila B., Chuaqui C., Hird A.W., Lamb M.L., Rawlins P.B., Su N., Tentarelli S., Grimster N.P. (2016). Inhibition of Mcl-1 through covalent modification of a noncatalytic lysine side chain. Nat. Chem. Biol..

[172] Sengupta P., Chattopadhyay S., Chatterjee S. (2017). G-Quadruplex surveillance in BCL-2 gene: A promising therapeutic intervention in cancer treatment. Drug Discov. Today.

[173] Onel B., Carver M., Wu G., Timonina D., Kalarn S., Larriva M., Yang D. (2016). A New G-Quadruplex with Hairpin Loop Immediately Upstream of the Human BCL2 P1 Promoter Modulates Transcription. J. Am. Chem. Soc..

[174] Shahid R., Bugaut A., Balasubramanian S. (2010). The BCL-2 5’ untranslated region contains an RNA G-quadruplex-forming motif that modulates protein expression. Biochemistry.

[175] Jana J., Mondal S., Bhattacharjee P., Sengupta P., Roychowdhury T., Saha P., Kundu P., Chatterjee S. (2017). Chelerythrine down regulates expression of VEGFA, BCL2 and KRAS by arresting G-Quadruplex structures at their promoter regions. Sci. Rep..

[176] Casagrande V., Salvati E., Alvino A., Bianco A., Ciammaichella A., D’Angelo C., Ginnari-Satriani L., Serrilli A.M., Iachettini S., Leonetti C. (2011). N-cyclic bay-substituted perylene G-quadruplex ligands have selective antiproliferative effects on cancer cells and induce telomere damage. J. Med. Chem..

[177] Zhang J., Yu Q., Li Q., Yang L., Chen L., Zhou Y., Liu J. (2014). A ruthenium(II) complex capable of inducing and stabilizing bcl-2 G-quadruplex formation as a potential cancer inhibitor. J. Inorg. Biochem..

[178] Pelliccia S., Amato J., Capasso D., Di Gaetano S., Massarotti A., Piccolo M., Irace C., Tron G.C., Pagano B., Randazzo A. (2020). Bio-Inspired Dual-Selective BCL-2/c-MYC G-Quadruplex Binders: Design, Synthesis, and Anticancer Activity of Drug-like Imidazo [2,1-i]purine Derivatives. J. Med. Chem..

[179] Amato J., Pagano A., Capasso D., Di Gaetano S., Giustiniano M., Novellino E., Randazzo A., Pagano B. (2018). Targeting the BCL2 Gene Promoter G-Quadruplex with a New Class of Furopyridazinone-Based Molecules. Chem. Med. Chem..

[180] Gunaratnam M., Collie G.W., Reszka A.P., Todd A.K., Parkinson G.N., Neidle S. (2018). A naphthalene diimide G-quadruplex ligand inhibits cell growth and down-regulates BCL-2 expression in an imatinib-resistant gastrointestinal cancer cell line. Bioorg. Med. Chem..

[181] Andersen M.H., Reker S., Kvistborg P., Becker J.C., thor Straten P. (2005). Spontaneous immunity against Bcl-xL in cancer patients. J. Immunol..

[182] Larsen H.L., Andersen M.H., Wandall H.H., Madsen C.B., Christensen R.E., Petersen T.R., Pedersen A.E. (2014). Induction of Bcl-xL-specific cytotoxic T lymphocytes in mice. Scand. J. Immunol..

[183] Jorgensen N.G., Ahmad S.M., Abildgaard N., Straten P.T., Svane I.M., Andersen M.H., Knudsen L.M. (2016). Peptide vaccination against multiple myeloma using peptides derived from anti-apoptotic proteins: A phase I trial. Stem Cell Investig..

[184] Robin A.Y., Krishna Kumar K., Westphal D., Wardak A.Z., Thompson G.V., Dewson G., Colman P.M., Czabotar P.E. (2015). Crystal structure of Bax bound to the BH3 peptide of Bim identifies important contacts for interaction. Cell Death Dis..

[185] Garner T.P., Reyna D.E., Priyadarshi A., Chen H.C., Li S., Wu Y., Ganesan Y.T., Malashkevich V.N., Cheng E.H., Gavathiotis E. (2016). An Autoinhibited Dimeric Form of BAX Regulates the BAX Activation Pathway. Mol. Cell.

[186] Garner T.P., Lopez A., Reyna D.E., Spitz A.Z., Gavathiotis E. (2017). Progress in targeting the BCL-2 family of proteins. Curr. Opin. Chem. Biol..

[187] Hadji A., Schmitt G.K., Schnorenberg M.R., Roach L., Hickey C.M., Leak L.B., Tirrell M.V., LaBelle J.L. (2019). Preferential targeting of MCL-1 by a hydrocarbon-stapled BIM BH3 peptide. Oncotarget.

[188] Schnorenberg M.R., Bellairs J.A., Samaeekia R., Acar H., Tirrell M.V., LaBelle J.L. (2019). Activating the Intrinsic Pathway of Apoptosis Using BIM BH3 Peptides Delivered by Peptide Amphiphiles with Endosomal Release. Materials (Basel).

[189] Chen Y., Wang J., Zhang J., Wang W. (2018). Binding modes of Bcl-2 homology 3 (BH3) peptides with anti-apoptotic protein A1 and redesign of peptide inhibitors: A computational study. J. Biomol. Struct. Dyn..

[190] Vila-Julia G., Granadino-Roldan J.M., Perez J.J., Rubio-Martinez J. (2020). Molecular Determinants for the Activation/Inhibition of Bak Protein by BH3 Peptides. J. Chem. Inf. Model..

[191] Banta K.L., Wang X., Das P., Winoto A. (2018). B cell lymphoma 2 (Bcl-2) residues essential for Bcl-2’s apoptosis-inducing interaction with Nur77/Nor-1 orphan steroid receptors. J. Biol. Chem..

[192] Kolluri S.K., Zhu X., Zhou X., Lin B., Chen Y., Sun K., Tian X., Town J., Cao X., Lin F. (2008). A short Nur77-derived peptide converts Bcl-2 from a protector to a killer. Cancer Cell.

