# Supplementary material for: Inhibition of Anti-Apoptotic Bcl-2 Proteins in Preclinical and Clinical Studies: Current Overview in Cancer
Source: Cells. 2020 May 21;9(5):1287. doi: 10.3390/cells9051287 (PMC7291206; doi:10.3390/cells9051287)
Supplement: Supplementary file 1 [file cells-09-01287-s001.zip › Supplementary tables in word/Supplementary Table 1.docx]

ClinicalTrials.gov Search Results 04/07/2020

|  | Title | Status | Study Results | Conditions | Interventions | Locations |
| --- | --- | --- | --- | --- | --- | --- |
| 1 | [A Phase I/II Study of Oblimersen Plus Cisplatin and](https://ClinicalTrials.gov/show/NCT00064259) [Fluorouracil in Gastric & Esophageal Junction Cancer](https://ClinicalTrials.gov/show/NCT00064259) | Terminated | Has Results | - Adenocarcinoma of the Esophagus - Adenocarcinoma of the Gastroesophageal Junction - Diffuse Adenocarcinoma of the Stomach - Intestinal Adenocarcinoma of the Stomach - Mixed Adenocarcinoma of the Stomach - Recurrent Esophageal Cancer - Recurrent Gastric Cancer - Squamous Cell Carcinoma of the Esophagus - Stage III Esophageal Cancer - Stage IIIA Gastric Cancer - and 4 more | - Biological: oblimersen sodium - Drug: cisplatin - Drug: fluorouracil | - Montefiore Medical Center, Bronx, New York, United States |
| 2 | [Pharmacokinetic Study of Genasense in Subjects With](https://ClinicalTrials.gov/show/NCT00543075) [Normal Renal Function, Mildly Impaired Renal Function, and](https://ClinicalTrials.gov/show/NCT00543075) [Moderately Impaired Renal Function](https://ClinicalTrials.gov/show/NCT00543075) | Completed | No Results Available | - Normal Renal Function - Mildly Impaired Renal Function - Moderately Impaired Renal Function | - Drug: Genasense (oblimersen, G3139) | - DaVita Clinical Research, Minneapolis, Minnesota, United States |
| 3 | [Rituximab and Oblimersen in Treating Patients With Stage II,](https://ClinicalTrials.gov/show/NCT00301795) [Stage III, or Stage IV Follicular Non-Hodgkin's Lymphoma](https://ClinicalTrials.gov/show/NCT00301795) | Terminated | No Results Available | - Stage III Grade 1 Follicular Lymphoma - Stage III Grade 2 Follicular Lymphoma - Stage III Grade 3 Follicular Lymphoma - Stage IV Grade 1 Follicular Lymphoma - Stage IV Grade 2 Follicular Lymphoma - Stage IV Grade 3 Follicular Lymphoma | - Biological: oblimersen sodium - Biological: rituximab - Other: laboratory biomarker analysis | - Cancer and Leukemia Group B, Chicago, Illinois, United States |
| 4 | [Pharmacokinetics of G3139 in Subjects With Advanced](https://ClinicalTrials.gov/show/NCT00543205) [Melanoma, Including Those With Normal Hepatic Function](https://ClinicalTrials.gov/show/NCT00543205) [and Those With Moderate Hepatic Impairment](https://ClinicalTrials.gov/show/NCT00543205) | Terminated | No Results Available | - Advanced Melanoma and Normal or Impaired - Hepatic Function | - Drug: Genasense® (G3139, oblimersen sodium) |  |
| 5 | [Oblimersen in Treating Patients With Relapsed or Refractory](https://ClinicalTrials.gov/show/NCT00062244) [Waldenstrom's Macroglobulinemia](https://ClinicalTrials.gov/show/NCT00062244) | Completed | No Results Available | - Waldenström Macroglobulinemia | - Biological: oblimersen sodium | - Mayo Clinic in Arizona, Scottsdale, Arizona, United States - Howard University Hospital, Washington, District of Columbia, United States - Mayo Clinic in Florida, Jacksonville, Florida, United States - University of Maryland Greenebaum Cancer Center, Baltimore, Maryland, United States - Johns Hopkins University, Baltimore, Maryland, United States - Barbara Ann Karmanos Cancer Institute, Detroit, Michigan, United States - Mayo Clinic, Rochester, Minnesota, United States - University of Wisconsin Hospital and Clinics, Madison, Wisconsin, United States |
| 6 | [Genasense as a 2-hour Intravenous Infusion in Subjects With](https://ClinicalTrials.gov/show/NCT00636545) [Solid Tumors](https://ClinicalTrials.gov/show/NCT00636545) | Completed | No Results Available | - Solid Tumors | - Drug: Oblimersen (Genasense) | - South Texas Accelerated Research Therapeutics, San Antonio, Texas, United States |

|  | Title | Status | Study Results | Conditions | Interventions | Locations |
| --- | --- | --- | --- | --- | --- | --- |
| 7 | [Combination Chemotherapy Plus Oblimersen in Treating](https://ClinicalTrials.gov/show/NCT00017251) [Patients With Previously Untreated Extensive-Stage Small](https://ClinicalTrials.gov/show/NCT00017251) [Cell Lung Cancer](https://ClinicalTrials.gov/show/NCT00017251) | Completed | No Results Available | - Extensive Stage Small Cell Lung Cancer | - Biological: oblimersen sodium - Drug: carboplatin - Drug: etoposide - Other: pharmacological study - Other: laboratory biomarker analysis | - University of Chicago Comprehensive Cancer Center, Chicago, Illinois, United States |
| 8 | [Oblimersen in Treating Patients With Merkel Cell Carcinoma](https://ClinicalTrials.gov/show/NCT00079131) | Completed | No Results Available | - Recurrent Neuroendocrine Carcinoma of the Skin - Stage I Neuroendocrine Carcinoma of the Skin - Stage II Neuroendocrine Carcinoma of the Skin - Stage III Neuroendocrine Carcinoma of the Skin - Stage IV Neuroendocrine Carcinoma of the Skin | - Biological: oblimersen sodium - Other: pharmacological study - Other: laboratory biomarker analysis | - Memorial Sloan-Kettering Cancer Center, New York, New York, United States |
| 9 | [Genasense, Carboplatin, Paclitaxel (GCP) Combination in](https://ClinicalTrials.gov/show/NCT01200342) [Uveal Melanoma](https://ClinicalTrials.gov/show/NCT01200342) | Terminated | Has Results | - Melanoma | - Drug: Genasense - Drug: Paclitaxel - Drug: Carboplatin | - UT MD Anderson Cancer Center, Houston, Texas, United States |
| 10 | [Oblimersen and Gemcitabine in Treating Patients With](https://ClinicalTrials.gov/show/NCT00060112) [Advanced Solid Tumor or Lymphoma](https://ClinicalTrials.gov/show/NCT00060112) | Terminated | No Results Available | - Extranodal Marginal Zone B-cell Lymphoma of Mucosa-associated Lymphoid Tissue - Nodal Marginal Zone B-cell Lymphoma - Recurrent Adult Burkitt Lymphoma - Recurrent Adult Diffuse Large Cell Lymphoma - Recurrent Adult Diffuse Mixed Cell Lymphoma - Recurrent Adult Diffuse Small Cleaved Cell Lymphoma - Recurrent Adult Hodgkin Lymphoma - Recurrent Adult Immunoblastic Large Cell Lymphoma - Recurrent Adult Lymphoblastic Lymphoma - Recurrent Adult T-cell Leukemia/ Lymphoma - and 24 more | - Biological: oblimersen sodium - Drug: gemcitabine hydrochloride - Other: pharmacological study - Other: laboratory biomarker analysis | - Stanford University, Stanford, California, United States |
| 11 | [Oblimersen and Imatinib Mesylate in Treating Patients With](https://ClinicalTrials.gov/show/NCT00091078) [Advanced Gastrointestinal Stromal Tumors That Cannot Be](https://ClinicalTrials.gov/show/NCT00091078) [Removed By Surgery](https://ClinicalTrials.gov/show/NCT00091078) | Terminated | No Results Available | - Gastrointestinal Stromal Tumor | - Biological: oblimersen sodium - Drug: imatinib mesylate - Other: laboratory biomarker analysis | - M D Anderson Cancer Center, Houston, Texas, United States |
| 12 | [Oblimersen and Imatinib Mesylate in Treating Patients With](https://ClinicalTrials.gov/show/NCT00049192) [Chronic Myelogenous Leukemia](https://ClinicalTrials.gov/show/NCT00049192) | Completed | No Results Available | - Chronic Myelogenous Leukemia, BCR- ABL1 Positive - Chronic Phase Chronic Myelogenous Leukemia - Relapsing Chronic Myelogenous Leukemia | - Biological: oblimersen sodium - Drug: imatinib mesylate - Other: laboratory biomarker analysis | - Cancer and Leukemia Group B, Chicago, Illinois, United States |

|  | Title | Status | Study Results | Conditions | Interventions | Locations |
| --- | --- | --- | --- | --- | --- | --- |
| 13 | [Combination Chemotherapy Plus Oblimersen in Treating](https://ClinicalTrials.gov/show/NCT00054548) [Patients With Advanced Solid Tumors](https://ClinicalTrials.gov/show/NCT00054548) | Completed | No Results Available | - Unspecified Adult Solid Tumor, Protocol Specific | - Biological: oblimersen sodium - Drug: paclitaxel - Other: laboratory biomarker analysis - Other: pharmacological study | - University of Wisconsin Hospital and Clinics, Madison, Wisconsin, United States |
| 14 | [Carboplatin and Etoposide With or Without Oblimersen](https://ClinicalTrials.gov/show/NCT00042978) [Sodium in Treating Patients With Extensive Stage Small Cell](https://ClinicalTrials.gov/show/NCT00042978) [Lung Cancer](https://ClinicalTrials.gov/show/NCT00042978) | Completed | No Results Available | - Extensive Stage Small Cell Lung Cancer - Recurrent Small Cell Lung Cancer | - Biological: oblimersen sodium - Drug: carboplatin - Drug: etoposide | - Cancer and Leukemia Group B, Chicago, Illinois, United States |
| 15 | [Genasense® (Oblimersen Sodium), Fludarabine, and](https://ClinicalTrials.gov/show/NCT00078234) [Rituximab in Subjects With Chronic Lymphocytic Leukemia](https://ClinicalTrials.gov/show/NCT00078234) | Completed | No Results Available | - Chronic Lymphocytic Leukemia | - Drug: Oblimersen-rituximab-fludarabine | - Georgetown University Medical Center/Lombardi Cancer Center, Washington, District of Columbia, United States - Roswell Park Cancer Institute, Buffalo, New York, United States - Long Island Jewish Medical Center, New Hyde Park, New York, United States |
| 16 | [Oblimersen Sodium and Rituximab in Treating Patients With](https://ClinicalTrials.gov/show/NCT00054639) [Recurrent B-cell Non-Hodgkin Lymphoma](https://ClinicalTrials.gov/show/NCT00054639) | Completed | Has Results | - Cutaneous B-cell Non-Hodgkin Lymphoma - Extranodal Marginal Zone B-cell Lymphoma of Mucosa-associated Lymphoid Tissue - Intraocular Lymphoma - Nodal Marginal Zone B-cell Lymphoma - Recurrent Adult Burkitt Lymphoma - Recurrent Adult Diffuse Large Cell Lymphoma - Recurrent Adult Diffuse Mixed Cell Lymphoma - Recurrent Adult Diffuse Small Cleaved Cell Lymphoma - Recurrent Adult Grade III Lymphomatoid Granulomatosis - Recurrent Adult Immunoblastic Large Cell Lymphoma - and 11 more | - Biological: oblimersen sodium - Biological: rituximab - Other: laboratory biomarker analysis | - M D Anderson Cancer Center, Houston, Texas, United States |
| 17 | [Oblimersen and Interferon Alfa in Treating Patients With](https://ClinicalTrials.gov/show/NCT00059813) [Metastatic Renal Cell Cancer](https://ClinicalTrials.gov/show/NCT00059813) | Completed | No Results Available | - Recurrent Renal Cell Cancer - Stage IV Renal Cell Cancer | - Biological: recombinant interferon alfa - Biological: oblimersen sodium - Other: pharmacological study | - City of Hope, Duarte, California, United States |

|  | Title | Status | Study Results | Conditions | Interventions | Locations |
| --- | --- | --- | --- | --- | --- | --- |
| 18 | [Trial of Dacarbazine With or Without Genasense in Advanced](https://ClinicalTrials.gov/show/NCT00518895) [Melanoma](https://ClinicalTrials.gov/show/NCT00518895) | Completed | No Results Available | - Melanoma | - Drug: dacarbazine plus Genasense - Drug: dacarbazine plus placebo | - University of South Alabama Hospital, Mitchell Cancer Institute, Mobile, Alabama, United States - San Diego Pacific Oncology and Hematology Associates Inc., Encinitas, California, United States - Redwood Regional Medical Group, Inc., Santa Rosa, California, United States - Siouxland Hematology Oncology Associates, Sioux City, Iowa, United States - Dana Farber Cancer Institute, Boston, Massachusetts, United States - Hematology Oncology Centers of the Northern Rockies, Billings, Montana, United States - Morristown Memorial - Atlantic Healthcare System, Morristown, New Jersey, United States - Cancer Care Associates, Oklahoma City, Oklahoma, United States - Cancer Care Associates, Site 1, Tulsa, Oklahoma, United States - St. Luke's Cancer Center, Bethlehem, Pennsylvania, United States - and 67 more |
| 19 | [Oblimersen Plus Doxorubicin and Docetaxel in Treating](https://ClinicalTrials.gov/show/NCT00063934) [Patients With Metastatic or Locally Advanced Breast Cancer](https://ClinicalTrials.gov/show/NCT00063934) | Terminated | Has Results | - Male Breast Cancer - Stage IIIA Breast Cancer - Stage IIIB Breast Cancer - Stage IIIC Breast Cancer - Stage IV Breast Cancer | - Biological: oblimersen sodium - Drug: doxorubicin hydrochloride - Drug: docetaxel - Biological: filgrastim - Biological: pegfilgrastim - Procedure: therapeutic conventional surgery - Other: pharmacological study - Other: laboratory biomarker analysis | - M D Anderson Cancer Center, Houston, Texas, United States |
| 20 | [Oblimersen, Cytarabine, and Daunorubicin in Treating Older](https://ClinicalTrials.gov/show/NCT00039117) [Patients With Acute Myeloid Leukemia](https://ClinicalTrials.gov/show/NCT00039117) | Completed | No Results Available | - Adult Acute Myeloid Leukemia With 11q23 (MLL) Abnormalities - Adult Acute Myeloid Leukemia With Inv(16) (p13;q22) - Adult Acute Myeloid Leukemia With t(16;16)(p13;q22) - Adult Acute Myeloid Leukemia With t(8;21) (q22;q22) - Secondary Acute Myeloid Leukemia - Untreated Adult Acute Myeloid Leukemia | - Biological: oblimersen sodium - Drug: cytarabine - Drug: daunorubicin hydrochloride - Other: laboratory biomarker analysis - Other: pharmacological study | - Ohio State University Medical Center, Columbus, Ohio, United States |

|  | Title | Status | Study Results | Conditions | Interventions | Locations |
| --- | --- | --- | --- | --- | --- | --- |
| 21 | [Daunorubicin Hydrochloride, Cytarabine and Oblimersen](https://ClinicalTrials.gov/show/NCT00085124) [Sodium in Treating Patients With Previously Untreated Acute](https://ClinicalTrials.gov/show/NCT00085124) [Myeloid Leukemia](https://ClinicalTrials.gov/show/NCT00085124) | Completed | No Results Available | - Adult Acute Myeloid Leukemia With 11q23 (MLL) Abnormalities - Adult Acute Myeloid Leukemia With Inv(16) (p13;q22) - Adult Acute Myeloid Leukemia With t(15;17)(q22;q12) - Adult Acute Myeloid Leukemia With t(16;16)(p13;q22) - Adult Acute Myeloid Leukemia With t(8;21) (q22;q22) - Secondary Acute Myeloid Leukemia - Untreated Adult Acute Myeloid Leukemia | - Biological: oblimersen sodium - Drug: cytarabine - Drug: daunorubicin hydrochloride - Other: laboratory biomarker analysis | - Cancer and Leukemia Group B, Chicago, Illinois, United States - Arthur G. James Cancer Hospital and Solove Research Institute at Ohio State University Medical Center, Columbus, Ohio, United States |
| 22 | [Oblimersen and Doxorubicin in Treating Patients With](https://ClinicalTrials.gov/show/NCT00047229) [Advanced Hepatocellular Carcinoma (Liver Cancer)](https://ClinicalTrials.gov/show/NCT00047229) | Completed | No Results Available | - Liver Cancer | - Biological: oblimersen sodium - Drug: doxorubicin hydrochloride | - British Columbia Cancer Agency - Vancouver Cancer Centre, Vancouver, British Columbia, Canada - London Regional Cancer Program at London Health Sciences Centre, London, Ontario, Canada - Princess Margaret Hospital, Toronto, Ontario, Canada |
| 23 | [This Study is Being Performed to Evaluate the Effect of](https://ClinicalTrials.gov/show/NCT00517218) [Genasense on the Efficacy and the Safety of Rituximab/](https://ClinicalTrials.gov/show/NCT00517218) [Fludarabine Combination Treatment in Previously Untreated](https://ClinicalTrials.gov/show/NCT00517218) [Subjects With Chronic Lymphocyctic Leukemia(CLL)](https://ClinicalTrials.gov/show/NCT00517218) | Withdrawn | No Results Available | - Lymphocytic Leukemia | - Drug: Genasense® (, oblimersen sodium G3139) |  |
| 24 | [Abraxane and Temodar Plus Genasense in Advanced](https://ClinicalTrials.gov/show/NCT00409383) [Melanoma](https://ClinicalTrials.gov/show/NCT00409383) | Unknown status | No Results Available | - Melanoma | - Drug: Genasense® (oblimersen) - Drug: Abraxane® (paclitaxel protein-bound particles for injectable suspension) - Drug: Temodar® (temozolomide) | - New York University Cancer Center, New York, New York, United States |
| 25 | [Docetaxel With or Without Oblimersen in Treating Patients](https://ClinicalTrials.gov/show/NCT00085228) [With Hormone-Refractory Adenocarcinoma (Cancer) of the](https://ClinicalTrials.gov/show/NCT00085228) [Prostate](https://ClinicalTrials.gov/show/NCT00085228) | Completed | No Results Available | - Prostate Cancer | - Biological: oblimersen sodium - Drug: docetaxel | - Kaiser Franz Josef Hospital, Vienna, Austria - Onze Lieve Vrouw Ziekenhuis Aalst, Aalst, Belgium - Institut Jules Bordet, Brussels, Belgium - Cliniques Universitaires Saint-Luc, Brussels, Belgium - Universitair Ziekenhuis Gent, Ghent, Belgium - U.Z. Gasthuisberg, Leuven, Belgium - Rigshospitalet - Copenhagen University Hospital, Copenhagen, Denmark - CHU de Grenoble - Hopital de la Tronche, Grenoble, France - Assaf Harofeh Medical Center, Zerifin, Israel - Ospedale S. Camillo-Forlanini, Rome, Italy - and 6 more |
| 26 | [Oblimersen and Dacarbazine in Treating Patients With](https://ClinicalTrials.gov/show/NCT00070343) [Advanced Malignant Melanoma That Has Responded to](https://ClinicalTrials.gov/show/NCT00070343) [Treatment on Clinical Trial GENTA-GM301](https://ClinicalTrials.gov/show/NCT00070343) | Unknown status | No Results Available | - Melanoma (Skin) | - Biological: oblimersen sodium - Drug: dacarbazine | - Jonsson Comprehensive Cancer Center, UCLA, Los Angeles, California, United States |
| 27 | [A Phase I, Randomized, Open-label, Cross-over,](https://ClinicalTrials.gov/show/NCT00542893) [Pharmacokinetic Study of Genasense With and Without](https://ClinicalTrials.gov/show/NCT00542893) [Dacarbazine](https://ClinicalTrials.gov/show/NCT00542893) | Completed | No Results Available | - Advanced Melanoma | - Drug: Genasense® (G3139, oblimersen sodium) - Drug: Genasense followed by DTIC; then DTIC alone - Drug: DTIC alone; then Genasense followed by DTIC |  |

|  | Title | Status | Study Results | Conditions | Interventions | Locations |
| --- | --- | --- | --- | --- | --- | --- |
| 28 | [Dacarbazine With or Without Oblimersen (G3139) in Treating](https://ClinicalTrials.gov/show/NCT00016263) [Patients With Advanced Malignant Melanoma](https://ClinicalTrials.gov/show/NCT00016263) | Completed | No Results Available | - Melanoma (Skin) | - Biological: oblimersen sodium - Drug: dacarbazine | - Jonsson Comprehensive Cancer Center, UCLA, Los Angeles, California, United States - Genta Incorporated, Berkeley Heights, New Jersey, United States |
| 29 | [Oblimersen and Gemtuzumab Ozogamicin in Treating Older](https://ClinicalTrials.gov/show/NCT00017589) [Patients With Relapsed Acute Myeloid Leukemia](https://ClinicalTrials.gov/show/NCT00017589) | Completed | No Results Available | - Leukemia | - Biological: oblimersen sodium - Drug: gemtuzumab ozogamicin | - Genta Incorporated, Berkeley Heights, New Jersey, United States |
| 30 | [Oblimersen Plus Combination Chemotherapy and](https://ClinicalTrials.gov/show/NCT00039481) [Dexrazoxane in Treating Children and Adolescents With](https://ClinicalTrials.gov/show/NCT00039481) [Relapsed or Refractory Solid Tumors](https://ClinicalTrials.gov/show/NCT00039481) | Completed | No Results Available | - Cardiac Toxicity - Unspecified Childhood Solid Tumor, Protocol Specific | - Biological: oblimersen sodium - Drug: dexrazoxane hydrochloride - Drug: doxorubicin hydrochloride - Drug: cyclophosphamide - Biological: filgrastim - Other: laboratory biomarker analysis - Other: pharmacological study | - Children's Oncology Group, Arcadia, California, United States |
| 31 | [Docetaxel With or Without Oblimersen in Treating Patients](https://ClinicalTrials.gov/show/NCT00030641) [With Non-Small Cell Lung Cancer](https://ClinicalTrials.gov/show/NCT00030641) | Unknown status | No Results Available | - Lung Cancer | - Biological: oblimersen sodium - Drug: docetaxel | - University of Alabama at Birmingham Comprehensive Cancer Center, Birmingham, Alabama, United States - Montgomery Cancer Center, Montgomery, Alabama, United States - Little Rock Hematology-Oncology Associates, Little Rock, Arkansas, United States - East Bay Medical Oncology, Concord, California, United States - Jonsson Comprehensive Cancer Center, UCLA, Los Angeles, California, United States - Medical Oncology Care Associates, Orange, California, United States - Pacific Hematology/Oncology, San Francisco, California, United States - John Wayne Cancer Institute at Saint John's Health Center, Santa Monica, California, United States - University of Colorado Cancer Center at University of Colorado Health Sciences Center, Aurora, Colorado, United States - Whittingham Cancer Center, Norwalk, Connecticut, United States - and 34 more |
| 32 | [Dexamethasone With or Without Oblimersen in Treating](https://ClinicalTrials.gov/show/NCT00017602) [Patients With Relapsed or Refractory Multiple Myeloma](https://ClinicalTrials.gov/show/NCT00017602) | Completed | No Results Available | - Multiple Myeloma and Plasma Cell Neoplasm | - Biological: oblimersen sodium - Drug: dexamethasone | - Genta Incorporated, Berkeley Heights, New Jersey, United States |
| 33 | [A Phase I Study of G3139 Subcutaneous in Solid Tumors](https://ClinicalTrials.gov/show/NCT00543231) | Completed | No Results Available | - Tumors | - Drug: G3139, Oblimersen sodium, Bcl-2 antisense oligonucleotide | - Clinical Research Institute for Drug Development, San Antonio, Texas, United States |

|  | Title | Status | Study Results | Conditions | Interventions | Locations |
| --- | --- | --- | --- | --- | --- | --- |
| 34 | [Bcl-2 Antisense Oligodeoxynucleotide G3139 and Paclitaxel in](https://ClinicalTrials.gov/show/NCT00005032) [Treating Patients With Recurrent Small Cell Lung Cancer](https://ClinicalTrials.gov/show/NCT00005032) | Completed | No Results Available | - Lung Cancer | - Biological: oblimersen sodium - Drug: paclitaxel | - University of Illinois at Chicago, Chicago, Illinois, United States - University of Chicago Cancer Research Center, Chicago, Illinois, United States - Louis A. Weiss Memorial Hospital, Chicago, Illinois, United States - Cancer Care Specialists of Central Illinois, S.C., Decatur, Illinois, United States - Evanston Northwestern Health Care, Evanston, Illinois, United States - Division of Hematology/Oncology, Park Ridge, Illinois, United States - Oncology/Hematology Associates of Central Illinois, P.C., Peoria, Illinois, United States - Central Illinois Hematology Oncology Center, Springfield, Illinois, United States - Fort Wayne Medical Oncology and Hematology, Inc., Fort Wayne, Indiana, United States - Michiana Hematology/Oncology P.C., South Bend, Indiana, United States - Arthur G. James Cancer Hospital - Ohio State University, Columbus, Ohio, United States |
| 35 | [Phase I/II Study of Genasense in Patients With Chronic](https://ClinicalTrials.gov/show/NCT00021749) [Lymphocytic Leukemia](https://ClinicalTrials.gov/show/NCT00021749) | Completed | No Results Available | - Chronic Lymphocytic Leukemia - CLL | - Drug: Oblimerson sodium, G3139 | - Long Island Jewish Medical Center, New Hyde Park, New York, United States - MD Anderson Cancer Center, Houston, Texas, United States - San Antonio Cancer Institute, San Antonio, Texas, United States |
| 36 | [Combination Chemotherapy and Oblimersen in Treating](https://ClinicalTrials.gov/show/NCT00055822) [Patients With Advanced Colorectal Cancer](https://ClinicalTrials.gov/show/NCT00055822) | Completed | No Results Available | - Colorectal Cancer | - Biological: oblimersen sodium - Drug: fluorouracil - Drug: leucovorin calcium - Drug: oxaliplatin | - San Antonio Cancer Institute, San Antonio, Texas, United States |
| 37 | [Oblimersen, Thalidomide, and Dexamethasone in Treating](https://ClinicalTrials.gov/show/NCT00049374) [Patients With Relapsed or Refractory Multiple Myeloma](https://ClinicalTrials.gov/show/NCT00049374) | Completed | No Results Available | - Multiple Myeloma and Plasma Cell Neoplasm | - Biological: oblimersen sodium - Drug: dexamethasone - Drug: thalidomide | - Greenebaum Cancer Center at University of Maryland Medical Center, Baltimore, Maryland, United States - St. Vincent's Comprehensive Cancer Center - Manhattan, New York, New York, United States |
| 38 | [S0349 Rituximab, Cyclophosphamide, Doxorubicin,](https://ClinicalTrials.gov/show/NCT00080847) [Vincristine, and Prednisone With or Without Oblimersen in](https://ClinicalTrials.gov/show/NCT00080847) [Treating Patients With Advanced Diffuse Large B-Cell Non-](https://ClinicalTrials.gov/show/NCT00080847) [Hodgkin's Lymphoma](https://ClinicalTrials.gov/show/NCT00080847) | Terminated | No Results Available | - Contiguous Stage II Adult Diffuse Large Cell Lymphoma - Noncontiguous Stage II Adult Diffuse Large Cell Lymphoma - Stage III Adult Diffuse Large Cell Lymphoma - Stage IV Adult Diffuse Large Cell Lymphoma | - Biological: oblimersen sodium - Biological: rituximab - Drug: cyclophosphamide - Drug: doxorubicin hydrochloride - Drug: vincristine sulfate - Drug: prednisone - Other: laboratory biomarker analysis | - Southwest Oncology Group, San Antonio, Texas, United States |

|  | Title | Status | Study Results | Conditions | Interventions | Locations |
| --- | --- | --- | --- | --- | --- | --- |
| 39 | [Oblimersen, Rituximab, Cyclophosphamide, Doxorubicin,](https://ClinicalTrials.gov/show/NCT00070083) [Vincristine, and Prednisone in Treating Patients With Stage II,](https://ClinicalTrials.gov/show/NCT00070083) [Stage III, or Stage IV Diffuse Large B-Cell Lymphoma](https://ClinicalTrials.gov/show/NCT00070083) | Completed | No Results Available | - Lymphoma | - Biological: oblimersen sodium - Biological: rituximab - Drug: cyclophosphamide - Drug: doxorubicin hydrochloride - Drug: prednisone - Drug: vincristine sulfate | - Stanford Cancer Center at Stanford University Medical Center, Stanford, California, United States - British Columbia Cancer Agency - Centre for the Southern Interior, Kelowna, British Columbia, Canada - Fraser Valley Cancer Centre at British Columbia Cancer Agency, Surrey, British Columbia, Canada - British Columbia Cancer Agency - Vancouver Cancer Centre, Vancouver, British Columbia, Canada |
| 40 | [Fludarabine and Cyclophosphamide With or Without](https://ClinicalTrials.gov/show/NCT00024440) [Oblimersen in Treating Patients With Relapsed or Refractory](https://ClinicalTrials.gov/show/NCT00024440) [Chronic Lymphocytic Leukemia](https://ClinicalTrials.gov/show/NCT00024440) | Completed | No Results Available | - Leukemia | - Biological: filgrastim - Biological: oblimersen sodium - Drug: cyclophosphamide - Drug: fludarabine phosphate | - Genta Incorporated, Berkeley Heights, New Jersey, United States |
| 41 | [Oblimersen Sodium and Combination Chemotherapy in](https://ClinicalTrials.gov/show/NCT00736450) [Treating Patients With Newly Diagnosed Stage I, Stage II,](https://ClinicalTrials.gov/show/NCT00736450) [Stage III, or Stage IV Diffuse Large B-Cell Lymphoma](https://ClinicalTrials.gov/show/NCT00736450) | Terminated | Has Results | - Contiguous Stage II Adult Diffuse Large Cell Lymphoma - Noncontiguous Stage II Adult Diffuse Large Cell Lymphoma - Stage I Adult Diffuse Large Cell Lymphoma - Stage III Adult Diffuse Large Cell Lymphoma - Stage IV Adult Diffuse Large Cell Lymphoma | - Biological: oblimersen sodium - Biological: rituximab - Drug: cyclophosphamide - Drug: doxorubicin hydrochloride - Drug: vincristine sulfate - Drug: prednisone - Procedure: biopsy - Genetic: microarray analysis - Other: immunohistochemistry staining method - Genetic: gene expression analysis - Genetic: cytogenetic analysis | - Saint Francis Medical Center, Grand Island, Nebraska, United States - UNMC Eppley Cancer Center at the University of Nebraska Medical Center, Omaha, Nebraska, United States |
| 42 | [Oblimersen, Rituximab and Combination Chemotherapy in](https://ClinicalTrials.gov/show/NCT00086944) [Treating Patients With Relapsed or Refractory Aggressive](https://ClinicalTrials.gov/show/NCT00086944) [Non-Hodgkin's Lymphoma](https://ClinicalTrials.gov/show/NCT00086944) | Completed | No Results Available | - Recurrent Adult Diffuse Large Cell Lymphoma - Recurrent Grade 3 Follicular Lymphoma - Recurrent Mantle Cell Lymphoma | - Biological: oblimersen sodium - Biological: rituximab - Drug: ifosfamide - Drug: carboplatin - Drug: etoposide - Biological: filgrastim - Biological: pegfilgrastim - Other: laboratory biomarker analysis | - University of Chicago Comprehensive Cancer Center, Chicago, Illinois, United States |
| 43 | [Augmerosen Plus Fludarabine and Cytarabine in Treating](https://ClinicalTrials.gov/show/NCT00004862) [Patients With Refractory or Relapsed Acute Myeloid Leukemia](https://ClinicalTrials.gov/show/NCT00004862) [or Acute Lymphoblastic Leukemia](https://ClinicalTrials.gov/show/NCT00004862) | Completed | No Results Available | - Leukemia | - Biological: filgrastim - Biological: oblimersen sodium - Drug: cytarabine - Drug: fludarabine phosphate | - Arthur G. James Cancer Hospital - Ohio State University, Columbus, Ohio, United States |

|  | Title | Status | Study Results | Conditions | Interventions | Locations |
| --- | --- | --- | --- | --- | --- | --- |
| 44 | [Chemotherapy in Treating Patients With Solid Tumors](https://ClinicalTrials.gov/show/NCT00003103) | Completed | No Results Available | - Bladder Cancer - Breast Cancer - Colorectal Cancer - Esophageal Cancer - Head and Neck Cancer - Kidney Cancer - Lung Cancer - Ovarian Cancer - Prostate Cancer - Unspecified Adult Solid Tumor, Protocol Specific | - Biological: oblimersen sodium - Drug: docetaxel | - Memorial Sloan-Kettering Cancer Center, New York, New York, United States |
| 45 | [Olimersen and Irinotecan in Treating Patients With Metastatic](https://ClinicalTrials.gov/show/NCT00004870) [or Recurrent Colorectal Cancer](https://ClinicalTrials.gov/show/NCT00004870) | Completed | No Results Available | - Colorectal Cancer | - Biological: oblimersen sodium - Drug: irinotecan hydrochloride | - Ireland Cancer Center, Cleveland, Ohio, United States - Brooke Army Medical Center, Fort Sam Houston, Texas, United States - San Antonio Cancer Institute, San Antonio, Texas, United States |

U.S. National Library of Medicine | U.S. National Institutes of Health | U.S. Department of Health & Human Services
