# Supplementary material for: Inhibition of Anti-Apoptotic Bcl-2 Proteins in Preclinical and Clinical Studies: Current Overview in Cancer
Source: Cells. 2020 May 21;9(5):1287. doi: 10.3390/cells9051287 (PMC7291206; doi:10.3390/cells9051287)
Supplement: Supplementary file 1 [file cells-09-01287-s001.zip › Supplementary tables in word/Supplementary Table 2.docx]

ClinicalTrials.gov Search Results 04/07/2020

|  | Title | Status | Study Results | Conditions | Interventions | Locations |
| --- | --- | --- | --- | --- | --- | --- |
| 1 | [Study of Obatoclax in Previously Untreated Acute Myeloid](https://ClinicalTrials.gov/show/NCT00684918) [Leukemia (AML)](https://ClinicalTrials.gov/show/NCT00684918) | Completed | No Results Available | - AML | - Drug: Obatoclax | - Northwestern University, Chicago, Illinois, United States - The University of Iowa, Iowa City, Iowa, United States - University of Kansas Medical Center Research Institute, Westwood, Kansas, United States - Dana Farber Cancer Institute, Boston, Massachusetts, United States - St. Joseph Mercy Hospital, Ann Arbor, Michigan, United States - University of Michigan Health System, Ann Arbor, Michigan, United States - Michigan State University - Breslin Cancer Center, Lansing, Michigan, United States - St. Vincent's Comprehensive Cancer Center, New York, New York, United States - Legacy Emanuel Hospital & Health Center, Portland, Oregon, United States - Penn State Hershey Cancer Institute-Clinical Trials Office, Hershey, Pennsylvania, United States - and 3 more |
| 2 | [A Phase I/II Study of GX15-070MS in Untreated CLL](https://ClinicalTrials.gov/show/NCT00600964) | Completed | No Results Available | - Chronic Lymphocytic Leukemia | - Drug: GX15-070MS | - University of CA- San Diego, La Jolla, California, United States - Lombardi Cancer Center, Washington, District of Columbia, United States - Milton S Hershey Medical Center Penn State Cancer Institute, Hershey, Pennsylvania, United States - UT MD Anderson Cancer Center, Houston, Texas, United States - Princess Margaret Hospital, Toronto, Ontario, Canada |
| 3 | [A Phase I/II Study of Carboplatin and Etoposide With or](https://ClinicalTrials.gov/show/NCT00682981) [Without Obatoclax in Extensive-stage Small Cell Lung Cancer](https://ClinicalTrials.gov/show/NCT00682981) [(ES-SCLC)](https://ClinicalTrials.gov/show/NCT00682981) | Completed | No Results Available | - Extensive-stage Small Cell Lung Cancer | - Drug: Obatoclax - Drug: Carboplatin/etoposide | - Clearview Cancer Institute, Huntsville, Alabama, United States - Northwest Alabama Cancer Center, Muscle Shoals, Alabama, United States - Mayo Clinic-Arizona, Scottsdale, Arizona, United States - Arizona Clinical Research Center, Tucson, Arizona, United States - City of Hope and Beckman Research Institute, Duarte, California, United States - University of California-San Diego Moores Cancer Center, LaJolla, California, United States - Georgetown University Hospital-Lombardi Comprehensive Cancer Center, Washington, District of Columbia, United States - Integrated Community Oncology Network, Jacksonville, Florida, United States - University of Miami-Sylvester Cancer Center, Miami, Florida, United States - Florida Cancer Institute, New Port Richey, Florida, United States - and 65 more |
| 4 | [Safety and Efficacy of Single Agent Obatoclax Mesylate](https://ClinicalTrials.gov/show/NCT00427856) [(GX15-070MS) Followed by a Combination With Rituximab for](https://ClinicalTrials.gov/show/NCT00427856) [Previously-untreated Follicular Lymphoma (FL)](https://ClinicalTrials.gov/show/NCT00427856) | Completed | No Results Available | - Lymphoma, Follicular | - Drug: Obatoclax mesylate - Drug: Rituximab | - The Cancer Center at Hackensack University Medical Center, Hackensack, New Jersey, United States |

|  | Title | Status | Study Results | Conditions | Interventions | Locations |
| --- | --- | --- | --- | --- | --- | --- |
| 5 | [Obatoclax and Bortezomib in Treating Patients With](https://ClinicalTrials.gov/show/NCT00538187) [Aggressive Relapsed or Recurrent Non-Hodgkin Lymphoma](https://ClinicalTrials.gov/show/NCT00538187) | Terminated | No Results Available | - Adult Non-Hodgkin Lymphoma - Recurrent Adult Diffuse Large Cell Lymphoma - Recurrent Grade 1 Follicular Lymphoma - Recurrent Grade 2 Follicular Lymphoma - Recurrent Grade 3 Follicular Lymphoma - Recurrent Mantle Cell Lymphoma - Recurrent Marginal Zone Lymphoma - Recurrent Small Lymphocytic Lymphoma | - Drug: obatoclax mesylate - Drug: bortezomib - Other: laboratory biomarker analysis - Other: pharmacological study | - City of Hope Medical Center, Duarte, California, United States |
| 6 | [Obatoclax for Systemic Mastocytosis](https://ClinicalTrials.gov/show/NCT00918931) | Terminated | Has Results | - Leukemia - Systemic Mastocytosis | - Drug: Obatoclax Mesylate | - UT MD Anderson Cancer Center, Houston, Texas, United States |
| 7 | [Safety and Efficacy of Obatoclax Mesylate (GX15-070MS) in](https://ClinicalTrials.gov/show/NCT00359892) [the Treatment of Hodgkin's Lymphoma](https://ClinicalTrials.gov/show/NCT00359892) | Completed | No Results Available | - Hodgkin's Lymphoma | - Drug: Obatoclax mesylate (GX15-070MS) | - MD Anderson Cancer Center, Houston, Texas, United States |
| 8 | [Safety and Efficacy of Obatoclax Mesylate (GX15-070MS) in](https://ClinicalTrials.gov/show/NCT00407303) [Combination With Bortezomib for the Treatment of Relapsed](https://ClinicalTrials.gov/show/NCT00407303) [or Refractory Mantle Cell Lymphoma (MCL)](https://ClinicalTrials.gov/show/NCT00407303) | Completed | No Results Available | - Mantle-Cell Lymphoma | - Drug: Obatoclax mesylate - Drug: Bortezomib | - NW Georgia Oncology Centers, Marietta, Georgia, United States - Northwestern University Feinberg School of Medicine, Chicago, Illinois, United States - Hackensack University Medical Center, Hackensack, New Jersey, United States - Roswell Park Medical Center, Buffalo, New York, United States - University of Wisconsin, Madison, Wisconsin, United States |
| 9 | [Obatoclax, Fludarabine, and Rituximab in Treating Patients](https://ClinicalTrials.gov/show/NCT00612612) [With Previously Treated Chronic Lymphocytic Leukemia](https://ClinicalTrials.gov/show/NCT00612612) | Terminated | No Results Available | - B-cell Chronic Lymphocytic Leukemia - Leukemia - Prolymphocytic Leukemia - Refractory Chronic Lymphocytic Leukemia - Stage I Chronic Lymphocytic Leukemia - Stage II Chronic Lymphocytic Leukemia - Stage III Chronic Lymphocytic Leukemia - Stage IV Chronic Lymphocytic Leukemia | - Drug: obatoclax mesylate - Drug: fludarabine phosphate - Biological: rituximab - Other: laboratory biomarker analysis | - Dana-Farber Cancer Institute, Boston, Massachusetts, United States |

|  | Title | Status | Study Results | Conditions | Interventions | Locations |
| --- | --- | --- | --- | --- | --- | --- |
| 10 | [Safety and Efficacy of Obatoclax Mesylate (GX15-070MS)for](https://ClinicalTrials.gov/show/NCT00413114) [the Treatment of Myelodysplastic Syndromes (MDS)](https://ClinicalTrials.gov/show/NCT00413114) | Completed | No Results Available | - Myelodysplastic Syndromes | - Drug: Obatoclax mesylate (GX15-070MS) | - Stanford University, Stanford, California, United States - Georgetown University Medical Center, Washington, District of Columbia, United States - James A. Haley Veterans Hospital, Tampa, Florida, United States - Emory University School of Medicine/ Winship Cancer Center, Atlanta, Georgia, United States - Northwest Georgia Oncology Centers, Marietta, Georgia, United States - University of Chicago, Chicago, Illinois, United States - University of Massachusetts Medical Center, Worcester, Massachusetts, United States - Michigan State University, Breslin Cancer Center CTO, Lansing, Michigan, United States - Hematology-Oncology Centers of the Northern Rockies, Billings, Montana, United States - St. Vincent's Comprehensive Cancer Center, New York, New York, United States - and 12 more |
| 11 | [Safety and Efficacy of Obatoclax Mesylate (GX15-070MS)in](https://ClinicalTrials.gov/show/NCT00405951) [Combination With Docetaxel for the Treatment of Non-Small](https://ClinicalTrials.gov/show/NCT00405951) [Cell Lung Cancer](https://ClinicalTrials.gov/show/NCT00405951) | Completed | No Results Available | - Lung Cancer | - Drug: Obatoclax mesylate 250 ml - Drug: Docetaxel | - Mayo Clinic College of Medicine, Scottsdale, Arizona, United States - Tower Oncology, Beverly Hills, California, United States - Georgetown University, Washington, District of Columbia, United States - H. Lee Moffitt Cancer Center, Tampa, Florida, United States - University of Maryland, Baltimore, Maryland, United States - MedStar Research Institute, Baltimore, Maryland, United States - Arlington Cancer Center, Arlington, Texas, United States |
| 12 | [Efficacy and Safety of Obatoclax Mesylate in Combination](https://ClinicalTrials.gov/show/NCT01563601) [With Carboplatin and Etoposide Compared With Carboplatin](https://ClinicalTrials.gov/show/NCT01563601) [and Etoposide Alone in Chemotherapy-Naive Patients With](https://ClinicalTrials.gov/show/NCT01563601) [Extensive-Stage Small Cell Lung Cancer](https://ClinicalTrials.gov/show/NCT01563601) | Withdrawn | No Results Available | - Extensive-stage Small Cell Lung Cancer | - Drug: Obatoclax Mesylate, Carboplatine and Etoposide - Drug: Carboplatin and Etoposide |  |
| 13 | [Obatoclax and Bortezomib in Treating Patients With Relapsed](https://ClinicalTrials.gov/show/NCT00719901) [or Refractory Multiple Myeloma](https://ClinicalTrials.gov/show/NCT00719901) | Terminated | Has Results | - Refractory Multiple Myeloma - Stage I Multiple Myeloma - Stage II Multiple Myeloma - Stage III Multiple Myeloma | - Drug: obatoclax mesylate - Drug: bortezomib - Other: laboratory biomarker analysis | - Mayo Clinic, Rochester, Minnesota, United States |

|  | Title | Status | Study Results | Conditions | Interventions | Locations |
| --- | --- | --- | --- | --- | --- | --- |
| 14 | [Obatoclax Mesylate, Vincristine Sulfate, Doxorubicin](https://ClinicalTrials.gov/show/NCT00933985) [Hydrochloride, and Dexrazoxane Hydrochloride in Treating](https://ClinicalTrials.gov/show/NCT00933985) [Young Patients With Relapsed or Refractory Solid Tumors,](https://ClinicalTrials.gov/show/NCT00933985) [Lymphoma, or Leukemia](https://ClinicalTrials.gov/show/NCT00933985) | Terminated | No Results Available | - Acute Leukemias of Ambiguous Lineage - Acute Undifferentiated Leukemia - Angioimmunoblastic T-cell Lymphoma - Blastic Phase Chronic Myelogenous Leukemia - Childhood Burkitt Lymphoma - Childhood Chronic Myelogenous Leukemia - Childhood Diffuse Large Cell Lymphoma - Childhood Immunoblastic Large Cell Lymphoma - Childhood Nasal Type Extranodal NK/T-cell Lymphoma - Cutaneous B-cell Non-Hodgkin Lymphoma - and 19 more | - Drug: dexrazoxane hydrochloride - Drug: doxorubicin hydrochloride - Drug: obatoclax mesylate - Drug: liposomal vincristine sulfate - Other: pharmacological study - Other: laboratory biomarker analysis | - University of Alabama at Birmingham, Birmingham, Alabama, United States - Childrens Hospital of Orange County, Orange, California, United States - Children's National Medical Center, Washington, District of Columbia, United States - Lurie Children's Hospital-Chicago, Chicago, Illinois, United States - Indiana University Medical Center, Indianapolis, Indiana, United States - Dana-Farber Cancer Institute, Boston, Massachusetts, United States - C S Mott Children's Hospital, Ann Arbor, Michigan, United States - University of Minnesota Medical Center-Fairview, Minneapolis, Minnesota, United States - Washington University School of Medicine, Saint Louis, Missouri, United States - Columbia University Medical Center, New York, New York, United States - and 10 more |
| 15 | [Obatoclax Mesylate and Topotecan Hydrochloride in Treating](https://ClinicalTrials.gov/show/NCT00521144) [Patients With Relapsed or Refractory Small Cell Lung Cancer](https://ClinicalTrials.gov/show/NCT00521144) [or Advanced Solid Tumors](https://ClinicalTrials.gov/show/NCT00521144) | Completed | Has Results | - Recurrent Small Cell Lung Cancer - Unspecified Adult Solid Tumor, Protocol Specific | - Drug: obatoclax mesylate - Drug: topotecan hydrochloride - Other: laboratory biomarker analysis | - Sidney Kimmel Comprehensive Cancer Center at Johns Hopkins Hospital, Baltimore, Maryland, United States - Memorial Sloan Kettering Cancer Center, New York, New York, United States |
| 16 | [Safety and Efficacy of Obatoclax Mesylate (GX15-070MS) in](https://ClinicalTrials.gov/show/NCT00360035) [the Treatment of Myelofibrosis With Myeloid Metaplasia](https://ClinicalTrials.gov/show/NCT00360035) | Completed | No Results Available | - Myelofibrosis | - Drug: Obatoclax mesylate (GX15-070MS) | - Georgetown University Medical Center, Washington, District of Columbia, United States - James A. Haley Veterans Hospital, Tampa, Florida, United States - Emory University, Atlanta, Georgia, United States - The University of Chicago, Chicago, Illinois, United States - University of Massachusetts Medical Center, Worcester, Massachusetts, United States - MD Anderson Cancer Center, Houston, Texas, United States - Princess Margaret Hospital, Toronto, Ontario, Canada |
| 17 | [Obatoclax Mesylate, Rituximab, and Bendamustine](https://ClinicalTrials.gov/show/NCT01238146) [Hydrochloride in Treating Patients With Relapsed or](https://ClinicalTrials.gov/show/NCT01238146) [Refractory Non-Hodgkin Lymphoma](https://ClinicalTrials.gov/show/NCT01238146) | Withdrawn | No Results Available | - Extranodal Marginal Zone B-cell Lymphoma of Mucosa-associated Lymphoid Tissue - Nodal Marginal Zone B-cell Lymphoma - Recurrent Grade 1 Follicular Lymphoma - Recurrent Grade 2 Follicular Lymphoma - Recurrent Mantle Cell Lymphoma - Recurrent Marginal Zone Lymphoma - Splenic Marginal Zone Lymphoma | - Drug: bendamustine hydrochloride - Drug: obatoclax mesylate - Biological: rituximab | - Arthur G. James Cancer Hospital and Solove Research Institute at Ohio State University Medical Center, Columbus, Ohio, United States |
| 18 | [Safety and Efficacy of Obatoclax Mesylate (GX15-070MS) for](https://ClinicalTrials.gov/show/NCT00438178) [the Treatment of Hematological Malignancies](https://ClinicalTrials.gov/show/NCT00438178) | Completed | No Results Available | - Hematological Malignancies | - Drug: Obatoclax mesylate (GX15-070MS) | - Georgetown University Medical Center, Washington, District of Columbia, United States - MD Anderson Cancer Center, Houston, Texas, United States - Princess Margaret Hospital, Toronto, Ontario, Canada |

|  | Title | Status | Study Results | Conditions | Interventions | Locations |
| --- | --- | --- | --- | --- | --- | --- |
| 19 | [A Phase I/II Study of GMX1777 in Combination With](https://ClinicalTrials.gov/show/NCT00724841) [Temozolomide for the Treatment of Metastatic Melanoma](https://ClinicalTrials.gov/show/NCT00724841) | Terminated | No Results Available | - Metastatic Melanoma | - Drug: Obatoclax Mesylate - Drug: Temozolomide | - University of Pennsylvania, Philadelphia, Pennsylvania, United States |
| 20 | [Obatoclax Mesylate in Samples From Young Patients With](https://ClinicalTrials.gov/show/NCT01150656) [Acute Myeloid Leukemia](https://ClinicalTrials.gov/show/NCT01150656) | Completed | No Results Available | - Leukemia | - Genetic: gene expression analysis - Genetic: microarray analysis - Genetic: protein expression analysis - Genetic: reverse transcriptase-polymerase chain reaction - Genetic: western blotting - Other: laboratory biomarker analysis - Other: pharmacological study |  |

U.S. National Library of Medicine | U.S. National Institutes of Health | U.S. Department of Health & Human Services
