# Supplementary material for: Inhibition of Anti-Apoptotic Bcl-2 Proteins in Preclinical and Clinical Studies: Current Overview in Cancer
Source: Cells. 2020 May 21;9(5):1287. doi: 10.3390/cells9051287 (PMC7291206; doi:10.3390/cells9051287)
Supplement: Supplementary file 1 [file cells-09-01287-s001.zip › Supplementary tables in word/Supplementary Table 3.docx]

**ClinicalTrials.gov Search Results 04/07/2020**

|  | Title | Status | Study Results | Conditions | Interventions | Locations |
| --- | --- | --- | --- | --- | --- | --- |
| 1 | [Tarceva and AT-101 for Patients With Advanced Non-Small](https://ClinicalTrials.gov/show/NCT00934076) [Cell Lung Cancer](https://ClinicalTrials.gov/show/NCT00934076) | Withdrawn | No Results Available | - Carcinoma, Non Small Cell Lung | - Drug: Tarceva plus AT-101 |  |
| 2 | [Erlotinib and AT-101 in Advanced Non-Small Cell Lung](https://ClinicalTrials.gov/show/NCT00988169) [Cancer (NSCLC) Patients With Epidermal Growth Factor](https://ClinicalTrials.gov/show/NCT00988169) [Receptor (EGFR) Activating Mutations](https://ClinicalTrials.gov/show/NCT00988169) | Terminated | Has Results | - Lung Cancer | - Drug: oral erlotinib and pulsed doses of oral AT-101 | - Memorial Sloan Kettering Cancer Center, New York, New York, United States |
| 3 | [An Open-label, Single-center, Phase 1/ 2 Study of](https://ClinicalTrials.gov/show/NCT00561197) [Chemoradiotherapy and AT-101 in Patients With Locally](https://ClinicalTrials.gov/show/NCT00561197) [Advanced Esophageal or Gastroesophageal Junction Cancer](https://ClinicalTrials.gov/show/NCT00561197) | Terminated | No Results Available | - Locally Advanced Esophageal or GE Junction Cancer | - Drug: AT-101 | - MD Anderson Cancer Center, Houston, Texas, United States |
| 4 | [Phase 2 Safety and Efficacy Study of AT-101 in Combination](https://ClinicalTrials.gov/show/NCT00286780) [With Rituximab in Patients With Chronic Lymphocytic](https://ClinicalTrials.gov/show/NCT00286780) [Leukemia](https://ClinicalTrials.gov/show/NCT00286780) | Completed | No Results Available | - Chronic Lymphocytic Leukemia | - Drug: AT-101 | - UCSD Moores Cancer Center, San Diego, California, United States |
| 5 | [Safety and Efficacy Study of AT-101 in Combination With](https://ClinicalTrials.gov/show/NCT00286793) [Docetaxel and Prednisone in Men With HRPC](https://ClinicalTrials.gov/show/NCT00286793) | Completed | No Results Available | - Prostate Cancer | - Drug: AT-101 | - Hot Springs, Arkansas, United States - Fort Meyers, Florida, United States - Chicago, Illinois, United States - Fridley, Minnesota, United States - Albuquerque, New Mexico, United States - Syracuse, New York, United States - Wilmington, North Carolina, United States - Portland, Oregon, United States - Hilton Head Island, South Carolina, United States - Germantown, Tennessee, United States - and 3 more |
| 6 | [Lenalidomide and AT-101 in Treating Patients With Relapsed](https://ClinicalTrials.gov/show/NCT01003769) [B-Cell Chronic Lymphocytic Leukemia](https://ClinicalTrials.gov/show/NCT01003769) | Active, not recruiting | No Results Available | - Recurrent Chronic Lymphocytic Leukemia | - Drug: Lenalidomide - Drug: R-(-)-Gossypol Acetic Acid - Other: Laboratory Biomarker Analysis | - Mayo Clinic in Florida, Jacksonville, Florida, United States - Roswell Park Cancer Institute, Buffalo, New York, United States |
| 7 | [A Study of AT-101 in Combination With Docetaxel in](https://ClinicalTrials.gov/show/NCT01285635) [Squamous Cell Carcinoma Of The Head and Neck](https://ClinicalTrials.gov/show/NCT01285635) | Terminated | Has Results | - Squamous Cell Carcinoma of the Head and Neck (SCCHN) | - Drug: AT-101 - Drug: Docetaxel | - University of Michigan Comprehensive Cancer Center, Ann Arbor, Michigan, United States - Barbara Ann Karmanos Cancer Institute, Detroit, Michigan, United States |
| 8 | [A Study Comparing AT-101 in Combination With Docetaxel](https://ClinicalTrials.gov/show/NCT00571675) [and Prednisone Versus Docetaxel and Prednisone in Men](https://ClinicalTrials.gov/show/NCT00571675) [With Chemotherapy-Naïve Metastatic Hormone Refractory](https://ClinicalTrials.gov/show/NCT00571675) [Prostate Cancer (HRPC)](https://ClinicalTrials.gov/show/NCT00571675) | Completed | No Results Available | - Hormone Refractory Prostate Cancer | - Drug: AT-101, prednisone and docetaxel - Drug: placebo, prednisone and docetaxel | - Colorado Springs, Colorado, United States - New Port Richey, Florida, United States - Ocoee, Florida, United States - Fishers, Indiana, United States - Burnsville, Minnesota, United States - Las Vegas, Nevada, United States - Albuquerque, New Mexico, United States - Las Cruces, New Mexico, United States - Raleigh, North Carolina, United States - Kettering, Ohio, United States - and 25 more |

|  | Title | Status | Study Results | Conditions | Interventions | Locations |
| --- | --- | --- | --- | --- | --- | --- |
| 9 | [A Randomized Phase 2 Study of AT-101 in Combination](https://ClinicalTrials.gov/show/NCT00544960) [With Docetaxel in Relapsed/Refractory Non-Small Cell Lung](https://ClinicalTrials.gov/show/NCT00544960) [Cancer](https://ClinicalTrials.gov/show/NCT00544960) | Completed | No Results Available | - Non-small Cell Lung Cancer | - Drug: AT-101 and docetaxel - Drug: placebo and docetaxel | - Research Site, Durham, North Carolina, United States - Research Site, Arkhangelsk, Russian Federation - Research Site, Chelyabinsk, Russian Federation - Research Site, Ekaterinburg, Russian Federation - Research Site, Kaliningrad, Russian Federation - Research Site, Kazan, Russian Federation - Research Site, Moscow, Russian Federation - Research Sites (4), Saint Petersburg, Russian Federation - Research Site, Samara, Russian Federation - Research Site, Stavropol, Russian Federation - and 8 more |
| 10 | [Chemotherapy AND Bcl-xL Inhibitor (AT-101) For Organ](https://ClinicalTrials.gov/show/NCT01633541) [Preservation In Adults With Advanced Laryngeal Cancer](https://ClinicalTrials.gov/show/NCT01633541) | Active, not recruiting | No Results Available | - Laryngeal Cancer | - Drug: AT-101 - Drug: Docetaxel - Drug: Cisplatin - Drug: Carboplatin | - University of Michigan Comprehensive Cancer Center, Ann Arbor, Michigan, United States |
| 11 | [Study of AT-101 in Combination With Topotecan in Relapsed/](https://ClinicalTrials.gov/show/NCT00397293) [Refractory Small Cell Lung Cancer](https://ClinicalTrials.gov/show/NCT00397293) | Completed | No Results Available | - Small Cell Lung Cancer | - Drug: AT-101 - Drug: topotecan | - Birmingham, Alabama, United States - Hot Springs, Arkansas, United States - Loma Linda, California, United States - Stamford, Connecticut, United States - Jacksonville, Florida, United States - Lake City, Florida, United States - Boston, Massachusetts, United States - Rochester, Minnesota, United States - Lebanon, New Hampshire, United States - High Point, North Carolina, United States - and 9 more |
| 12 | [Gossypol Acetic Acid in Treating Patients With Recurrent,](https://ClinicalTrials.gov/show/NCT00848016) [Metastatic, or Primary Adrenocortical Cancer That Cannot Be](https://ClinicalTrials.gov/show/NCT00848016) [Removed By Surgery](https://ClinicalTrials.gov/show/NCT00848016) | Completed | Has Results | - Recurrent Adrenocortical Carcinoma - Stage III Adrenocortical Carcinoma - Stage IV Adrenocortical Carcinoma | - Drug: R-(-)-gossypol acetic acid | - University of Southern California, Los Angeles, California, United States - Mayo Clinic, Rochester, Minnesota, United States |

|  | Title | Status | Study Results | Conditions | Interventions | Locations |
| --- | --- | --- | --- | --- | --- | --- |
| 13 | [Safety & Efficacy Study of AT-101 in Combination w/](https://ClinicalTrials.gov/show/NCT00440388) [Rituximab in Previously Untreated Grade I-II Follicular Non-](https://ClinicalTrials.gov/show/NCT00440388) [Hodgkin's Lymphoma](https://ClinicalTrials.gov/show/NCT00440388) | Completed | No Results Available | - Follicular Lymphoma | - Drug: AT-101 - Drug: Rituximab | - University of Alabama at Birmingham, Birmingham, Alabama, United States - Hematology Oncology Associates, Phoenix, Arizona, United States - Rocky Mountain Cancer Center-Aurora, Aurora, Colorado, United States - Florida Cancer Institute, Hudson, Florida, United States - Florida Cancer Institute, New Port Richey, Florida, United States - Cancer Care & Hematology Specialists of Chicagoland, Arlington Heights, Illinois, United States - Central Indiana Cancer Centers, Fishers, Indiana, United States - University of Michigan Cancer Center, Ann Arbor, Michigan, United States - Minnesota Oncology Hematology, P.A., Minneapolis, Minnesota, United States - Missouri Cancer Associates, Columbia, Missouri, United States - and 18 more |
| 14 | [Phase II Safety and Efficacy Study of Single-agent AT-101 in](https://ClinicalTrials.gov/show/NCT00275431) [Patients With Relapsed or Refractory B-cell Malignancies](https://ClinicalTrials.gov/show/NCT00275431) | Completed | No Results Available | - Follicular Lymphoma - Diffuse Large Cell Lymphoma - Mantle Cell Lymphoma - Small Lymphocytic Lymphoma - Chronic Lymphocytic Leukemia | - Drug: AT-101 | - Birmingham, Alabama, United States - San Diego, California, United States - Atlanta, Georgia, United States - Chicago, Illinois, United States - Boston, Massachusetts, United States - New York, New York, United States - Rochester, New York, United States - High Point, North Carolina, United States - Hilton Head Island, South Carolina, United States - Memphis, Tennessee, United States - Burlington, Vermont, United States |
| 15 | [A Study of Single-Agent AT-101 in Men With Hormone](https://ClinicalTrials.gov/show/NCT00286806) [Refractory Prostate Cancer](https://ClinicalTrials.gov/show/NCT00286806) | Completed | No Results Available | - Hormone Refractory Prostate Cancer | - Drug: AT-101 | - Greenbrae, California, United States - New Haven, Connecticut, United States - Memphis, Tennessee, United States - Madison, Wisconsin, United States |
| 16 | [R-(-)-Gossypol Acetic Acid With Lenalidomide and](https://ClinicalTrials.gov/show/NCT02697344) [Dexamethasone in Treating Patients With Relapsed](https://ClinicalTrials.gov/show/NCT02697344) [Symptomatic Multiple Myeloma](https://ClinicalTrials.gov/show/NCT02697344) | Active, not recruiting | No Results Available | - Recurrent Plasma Cell Myeloma | - Drug: Dexamethasone - Other: Laboratory Biomarker Analysis - Drug: Lenalidomide - Other: Pharmacological Study - Drug: R-(-)-Gossypol Acetic Acid | - Mayo Clinic in Florida, Jacksonville, Florida, United States |

|  | Title | Status | Study Results | Conditions | Interventions | Locations |
| --- | --- | --- | --- | --- | --- | --- |
| 17 | [Gossypol in Treating Patients With Progressive or Recurrent](https://ClinicalTrials.gov/show/NCT00540722) [Glioblastoma Multiforme](https://ClinicalTrials.gov/show/NCT00540722) | Completed | Has Results | - Adult Giant Cell Glioblastoma - Adult Glioblastoma - Adult Gliosarcoma - Recurrent Adult Brain Tumor | - Drug: R-(-)-gossypol acetic acid - Other: laboratory biomarker analysis | - University of Alabama at Birmingham, Birmingham, Alabama, United States - Moffitt Cancer Center, Tampa, Florida, United States - Johns Hopkins University, Baltimore, Maryland, United States - Massachusetts General Hospital, Boston, Massachusetts, United States - Henry Ford Hospital, Detroit, Michigan, United States - Wake Forest University Health Sciences, Winston-Salem, North Carolina, United States - Cleveland Clinic Foundation, Cleveland, Ohio, United States - University of Pennsylvania, Philadelphia, Pennsylvania, United States |
| 18 | [R-(-)-Gossypol Acetic Acid, Cisplatin, and Etoposide in](https://ClinicalTrials.gov/show/NCT00544596) [Treating Patients With Advanced Solid Tumors or Extensive](https://ClinicalTrials.gov/show/NCT00544596) [Stage Small Cell Lung Cancer](https://ClinicalTrials.gov/show/NCT00544596) | Completed | No Results Available | - Extensive Stage Small Cell Lung Cancer - Unspecified Adult Solid Tumor, Protocol Specific | - Drug: R-(-)-gossypol acetic acid - Drug: cisplatin - Drug: etoposide | - Sanford Cancer Center-Oncology Clinic, Sioux Falls, South Dakota, United States - Gundersen Lutheran, La Crosse, Wisconsin, United States - UW Health Oncology - 1 South Park, Madison, Wisconsin, United States - University of Wisconsin Hospital and Clinics, Madison, Wisconsin, United States |
| 19 | [R-(-)-Gossypol Acetic Acid in Treating Patients With Recurrent](https://ClinicalTrials.gov/show/NCT00773955) [Extensive-Stage Small Cell Lung Cancer](https://ClinicalTrials.gov/show/NCT00773955) | Completed | Has Results | - Extensive Stage Small Cell Lung Cancer - Recurrent Small Cell Lung Cancer | - Drug: R-(-)-gossypol acetic acid - Other: pharmacological study - Other: laboratory biomarker analysis | - Mayo Clinic, Rochester, Minnesota, United States |
| 20 | [R-(-)-Gossypol and Androgen Ablation Therapy in Treating](https://ClinicalTrials.gov/show/NCT00666666) [Patients With Newly Diagnosed Metastatic Prostate Cancer](https://ClinicalTrials.gov/show/NCT00666666) | Completed | Has Results | - Adenocarcinoma of the Prostate - Stage IV Prostate Cancer | - Drug: AT-101 - Drug: Bicalutamide - Other: LHRH agent | - University of Chicago, Chicago, Illinois, United States - University of Michigan, Ann Arbor, Michigan, United States - Cancer Institute of New Jersey, New Brunswick, New Jersey, United States - University of Wisconsin Hospital and Clinics, Madison, Wisconsin, United States |

|  | Title | Status | Study Results | Conditions | Interventions | Locations |
| --- | --- | --- | --- | --- | --- | --- |
| 21 | [Gossypol, Paclitaxel, and Carboplatin in Treating Patients](https://ClinicalTrials.gov/show/NCT00891072) [With Solid Tumors That Are Metastatic or Cannot Be](https://ClinicalTrials.gov/show/NCT00891072) [Removed by Surgery](https://ClinicalTrials.gov/show/NCT00891072) | Completed | No Results Available | - Adult Grade III Lymphomatoid Granulomatosis - Adult Nasal Type Extranodal NK/T-cell Lymphoma - Contiguous Stage II Adult Burkitt Lymphoma - Contiguous Stage II Adult Diffuse Large Cell Lymphoma - Contiguous Stage II Adult Diffuse Mixed Cell Lymphoma - Contiguous Stage II Adult Diffuse Small Cleaved Cell Lymphoma - Contiguous Stage II Adult Immunoblastic Large Cell Lymphoma - Contiguous Stage II Adult Lymphoblastic Lymphoma - Contiguous Stage II Grade 1 Follicular Lymphoma - Contiguous Stage II Grade 2 Follicular Lymphoma - and 71 more | - Drug: R-(-)-gossypol acetic acid - Drug: paclitaxel - Drug: carboplatin - Other: pharmacological study | - Cancer Institute of New Jersey, New Brunswick, New Jersey, United States |
| 22 | [Gossypol (AT-101) and Temozolomide With or Without](https://ClinicalTrials.gov/show/NCT00390403) [Radiation Therapy in Treating Patients With Newly Diagnosed](https://ClinicalTrials.gov/show/NCT00390403) [Glioblastoma Multiforme](https://ClinicalTrials.gov/show/NCT00390403) | Completed | No Results Available | - Brain and Central Nervous System Tumors | - Drug: R-(-)-gossypol acetic acid - Drug: temozolomide - Genetic: gene expression analysis - Genetic: mutation analysis - Genetic: protein expression analysis - Other: laboratory biomarker analysis - Other: pharmacological study - Procedure: adjuvant therapy - Radiation: radiation therapy | - Lurleen Wallace Comprehensive Cancer at University of Alabama - Birmingham, Birmingham, Alabama, United States - H. Lee Moffitt Cancer Center and Research Institute at University of South Florida, Tampa, Florida, United States - Winship Cancer Institute of Emory University, Atlanta, Georgia, United States - Sidney Kimmel Comprehensive Cancer Center at Johns Hopkins, Baltimore, Maryland, United States - Josephine Ford Cancer Center at Henry Ford Hospital, Detroit, Michigan, United States - Wake Forest University Comprehensive Cancer Center, Winston-Salem, North Carolina, United States - Cleveland Clinic Taussig Cancer Center, Cleveland, Ohio, United States - Abramson Cancer Center of the University of Pennsylvania, Philadelphia, Pennsylvania, United States |
| 23 | [Gossypol Combined With Docetaxel and Cisplatin Scheme](https://ClinicalTrials.gov/show/NCT01977209) [in Advanced Non Small-cell Lung Cancers With APE1 High](https://ClinicalTrials.gov/show/NCT01977209) [Expression](https://ClinicalTrials.gov/show/NCT01977209) | Unknown status | No Results Available | - Non-small Cell Lung Cancer | - Drug: Gossypol - Drug: Placebo | - Daping Hospital, Third Military Medical University, Chongqing, Chongqing, China - Chongqing Zhongshan Hospital, Chongqing, Chongqing, China - Fuling Central Hospital, Chongqing, Chongqing, China - Jiangjin Central Hospital, Chongqing, Chongqing, China - The Second Affiliated Hospital of Medical University Of Chongqing, Chongqing, Chongqing, China - Three Gorges Central Hospital, Chongqing, Chongqing, China |
| 24 | [Investigation of AAI101 Safety, Tolerability & PK in Healthy](https://ClinicalTrials.gov/show/NCT03685084) [Volunteers](https://ClinicalTrials.gov/show/NCT03685084) | Completed | No Results Available | - Healthy | - Drug: AAI101 i.v. - Drug: Saline 0.9% infusion - Drug: Piperacillin i.v. - Drug: Cefepime i.v. |  |

|  | Title | Status | Study Results | Conditions | Interventions | Locations |
| --- | --- | --- | --- | --- | --- | --- |
| 25 | [A Study of DCR-A1AT in Healthy Adult Volunteers and](https://ClinicalTrials.gov/show/NCT04174118) [Patients With A1ATD-Associated Liver Disease](https://ClinicalTrials.gov/show/NCT04174118) | Recruiting | No Results Available | - Alpha 1-Antitrypsin Deficiency | - Drug: DCR-A1AT - Drug: Placebo | - Clinical Trial Consultants AB, Uppsala, Sweden |
| 26 | [Gastric Acid Rebound Secretion Measured by Alkaline Tide](https://ClinicalTrials.gov/show/NCT01315444) | Withdrawn | No Results Available | - Condition Measuring Alkaline Tide and Filling Symptoms' Questionnaire After Abrupt or Gradual Step Down Cessation of PPI | - Drug: Stop PPI gradually |  |
| 27 | [Study of the Safety, Tolerability, Pharmacokinetics and](https://ClinicalTrials.gov/show/NCT01265199) [Pharmacodynamic Properties of Oral AT-406 in Combination](https://ClinicalTrials.gov/show/NCT01265199) [With Daunorubicin and Cytarabine in Patients With Poor-risk](https://ClinicalTrials.gov/show/NCT01265199) [Acute Myelogenous Leukemia (AML)](https://ClinicalTrials.gov/show/NCT01265199) | Terminated | No Results Available | - Acute Myelogenous Leukemia (AML) | - Drug: AT-406 in combination with daunorubicin and cytarabine | - Univerity of Chicago, Chicago, Illinois, United States - University of Michigan Health System, Ann Arbor, Michigan, United States - Washington University at St. Louis Siteman Cancer Center, St. Louis, Missouri, United States - Memorial Sloan Kettering Cancer Center, New York, New York, United States - Hospital of the University of Pennsylvania, Philadelphia, Pennsylvania, United States - Temple University at Jeanes Hospital, Philadelphia, Pennsylvania, United States |
| 28 | [A Randomized Trial of Vaccine Adherence in Young Injection](https://ClinicalTrials.gov/show/NCT00244374) [Drug Users](https://ClinicalTrials.gov/show/NCT00244374) | Completed | Has Results | - Medication Adherence - Substance Abuse, Intravenous - Risk Behavior - Hepatitis A - Hepatitis B - Hepatitis C | - Biological: Hepatitis A & B vaccine - Behavioral: Outreach - Behavioral: AIC - Behavioral: SEP | - University of California San Francisco Medical Cen, San Francisco, California, United States |

U.S. National Library of Medicine | U.S. National Institutes of Health | U.S. Department of Health & Human Services
